# Common transcriptional changes in the brain during hibernation and Alzheimer’s disease

**DOI:** 10.1126/sciadv.aeg3486

**Published:** 2026-11-04

**Authors:** Pablo Largo-Barrientos, Roman Praschberger, Gonzalo León-Espinosa, Pieter-Jan Bultynck, Anastasia Mallopoulou, João F. Machado, Suresh Poovathingal, Kristofer Davie, Patrik Verstreken

**Affiliations:** ^1^ VIB-KU Leuven Center for Neuroscience; Leuven, Belgium.; ^2^ KU Leuven, Department of Neurosciences, Leuven Brain Institute, Mission Lucidity; Leuven, Belgium.; ^3^ Medical University of Innsbruck, Institute of Human Genetics; Innsbruck, Austria.; ^4^ Universidad San Pablo-Centro de Estudios Universitarios, Facultad de Farmacia, Departamento de Química y Bioquímica; Madrid, Spain.

## Abstract

Hibernation demands rapid brain adaptations, involving cycles of reduced metabolic rate during torpor, followed by brief periods of arousal. Previous studies show hibernation induces a drastic increase in Tau hyperphosphorylation, but a mechanistic connection to disease remains unclear. Using single-nucleus RNA sequencing on hippocampi of golden hamsters, we unveil large transcriptional shifts across the hibernation cycle in all cell types, especially glia. Common transcriptional changes in mitochondrial and ribosomal genes are observed in all different cell types. In addition, hibernation triggers an inflammatory-like response in microglia that in part resembles cell states observed in Alzheimer’s disease, but here in the absence of Aβ-accumulation and neurodegeneration. These findings reveal brain transformations that might help preserve vital functions under extreme conditions and they suggest that early cellular changes in AD may be a natural adaptive process.

## INTRODUCTION

Several animal species, including some primates (*1*, *2*), demonstrate the remarkable capability of hibernation, a condition where they significantly diminish bodily functions to preserve energy over extended periods of external adverse conditions. Hibernators often have distant phylogenetic origins, suggesting the existence of a shared, evolutionary-conserved genetic basis for hibernation mechanisms (*3*). This intriguing possibility raises the prospect that such genetic underpinnings might still exist in a latent form within non-hibernating species, including humans. Interestingly, archeological evidence found in hominin bones from Atapuerca suggests that human ancestors living half a million years ago underwent a hibernation-like hypometabolic state during extreme glaciation periods (*4*).

Hibernation is characterized by the oscillation between two phases: “torpor,” marked by immobility, reduced body temperature, and lowered cardiac, respiratory, and metabolic rates, and “arousal” periods, during which animals restore their physiological functions. The cyclic shift between torpor and arousal necessitates swift adaptations at the molecular, cellular, and tissue levels, aspects which have partially been revealed through pioneering histological and biochemical research (*5*–*7*). The discovery of well-controlled reversible metabolic and structural alterations has led to the idea that hibernation-linked changes are governed (at least in part) by differential gene expression (*8*, *9*). However, the analysis of cell-specific transcriptional changes has been hampered by limitations in sequencing resolution, sensitivity, and throughput.

In the brain, hibernation induces hyperphosphorylation of Tau (*10*), particularly in the hippocampus. Several phosphorylation sites of Tau observed in hibernation are intimately linked to the development of numerous forms of dementia, most notably Alzheimer’s disease (AD) (*11*). Interestingly, while Tau phosphorylation relentlessly progresses in disease leading to cognitive decline and neurodegeneration, in hibernation this is a physiological process that is fast and reversible and does not cause any brain damage (*10*). We hypothesize that hibernation and AD induce similar (as well as unique) molecular and cellular mechanisms in the brain. Here, we conducted single-nucleus RNA sequencing on the hippocampus from golden hamsters across the hibernation cycle and unveil large hibernation-induced transcriptional changes in all cell types, particularly in glia. We define hibernating microglial cell states and find striking similarities with some inflammatory states previously identified in AD patients, suggesting that these states represent a conserved and protective stress response.

## RESULTS

### Single-cell atlas of the golden hamster hippocampus

To define cellular state changes in the brain during hibernation, we induced this process in golden hamsters by placing them in a hibernaculum, a controlled environment that progressively reduced the temperature (from 23 to 4°C) and light hours (from 12 to 0). Analysis of the activity reported by movement detectors enabled identification of torpor and arousal phases (Fig. 1A). We euthanized animals in torpor and arousal as well as euthermic controls in three different experimental repeats (Fig. 1B), dissected the hippocampus, prepared a single-nuclei suspension, and used 10X Genomics single-nucleus RNA sequencing to reveal the nuclear transcriptome of around 100,000 cells. We verified the quality and homogeneity of the samples and the absence of batch effects (fig. S1). Computational analysis of this high-quality dataset resulted in the first single-cell resolution atlas of the golden hamster hippocampus during the hibernation cycle (Fig. 1C).

**Fig. 1.**
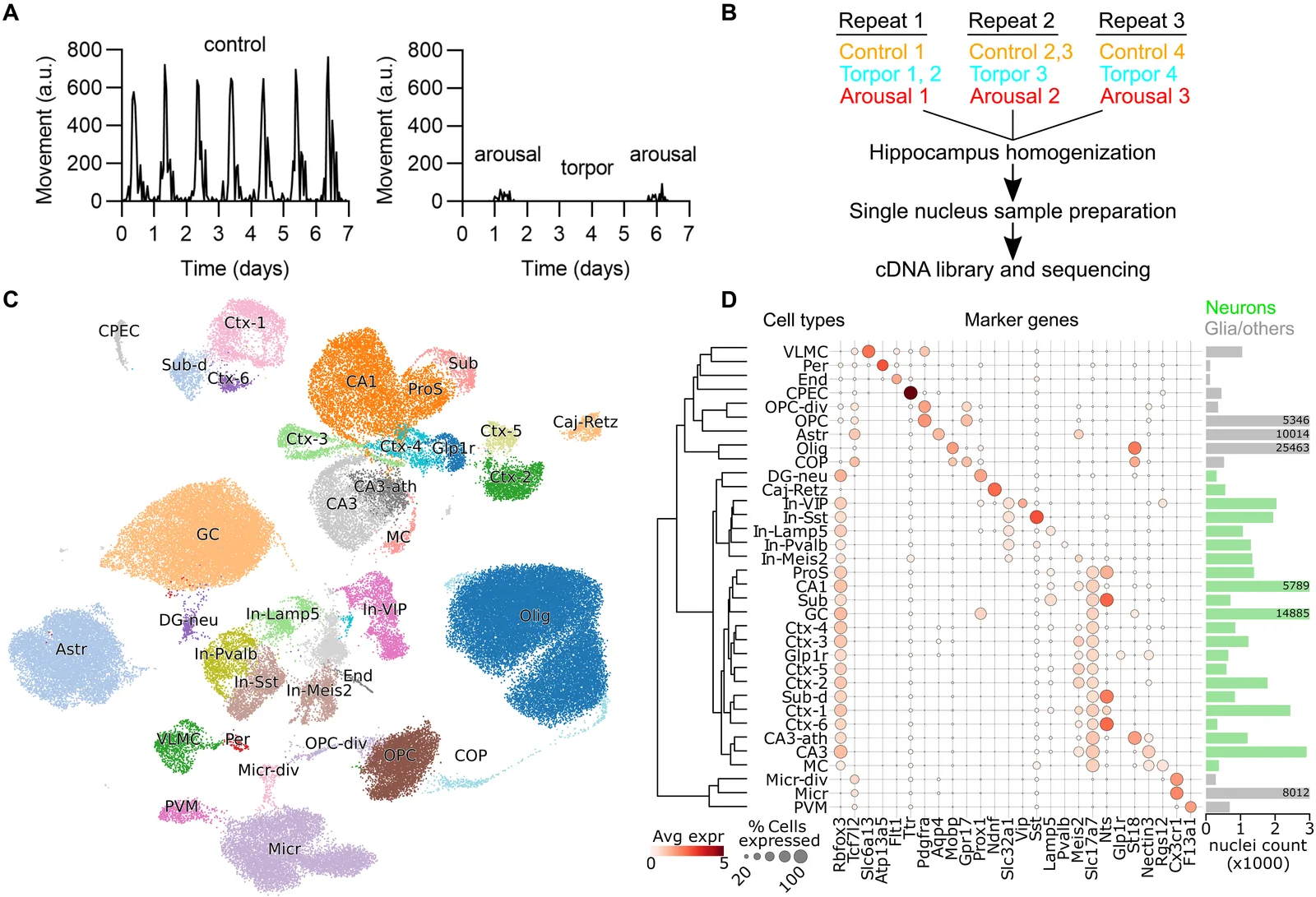
A single-cell atlas of the hibernating hamster hippocampus. (**A**) Graphs depicting movement (in arbitrary units) of golden hamsters as reported by the activity reporters located in their cages. Graph on the left depicts a representative movement pattern over 7 days for a control hamster housed at 23°C and normal 12:12 hours light/dark cycle. Graph on the right depicts a representative movement pattern over 7 days for a hamster housed at 4°C and complete darkness, transitioning over torpor and arousal phases. (**B**) Schematic representation of the experimental setup of the single nucleus RNA sequencing, with three separate repeats performed on different days and with all of them containing at least one animal from each of the three groups (control, torpor, arousal). (**C**) UMAP plot depicting around 100,000 cells from the hippocampi of control, torpor and arousal hamsters, colored based on the 34 different cell types that were identified (Astr, astrocytes; Caj-Retz, Cajal-Retzius cells; CA1, CA1 pyramidal neurons; CA3, CA3 pyramidal neurons; CA3-ath, CA3 athorny pyramidal neurons; COP, committed oligodendrocyte precursor cells; CPEC, choroid plexus epithelial cells; Ctx-1 to Ctx-6, cortical neurons; DG-neu, neuronal progenitor cells or immature granule cells from the subgranular zone of the dentate gyrus; End, endothelial cells; GC, dentate gyrus granule cells; Glp1r, Glp1r-expressing neurons; In-Lamp5, Lamp5 interneurons; In-Meis2, Meis2 interneurons; In-Pvalb, Parvalbumin interneurons; In-Sst, Somatostatin interneurons; In-VIP, VIP interneurons; MC, mossy cells; Micr, Microglia; Micr-div, dividing microglia; Olig, oligodendrocytes; OPC, oligodendrocyte precursor cells; OPC-div, dividing oligodendrocyte precursor cells; Per, pericytes; PVM, perivascular macrophages; ProS, prosubiculum; Sub, subiculum; Sub-d, dorsal subiculum; VLMC, vascular and lepotomeningeal cells). (**D**) Dendrogram showing the hierarchical organization of the 34 identified cell type clusters, the number of nuclei per cluster, and the average expression and percentage of cells expressing the main markers from each cluster.

Using a combination of automated and manual methods based on the expression of cell type-specific markers, we annotated 34 clusters that unequivocally represented different cell types (Fig. 1, C and D). Neurons (marked by *Rbfox3*) clustered in complex heterogenous groups whereas non-neuronal cell types (marked by *Tcf7l2*) formed more homogenous and defined clusters in the UMAP (fig. S2, A and B). Among the latter, we identified three major glial clusters: oligodendrocytes (marked by *Plp1*), astrocytes (marked by *Aqp4*) and microglia (marked by *Cx3cr1*) (fig. S2, C to E) as well as other smaller glial and non-glial cell types (fig. S2, F to O). Among the neurons, inhibitory GABAergic interneurons (marked by *Slc32a1*) and glutamatergic excitatory neurons (marked by *Slc17a7*) clearly separated from each other (fig. S2, P and Q). We identified the three main hippocampal excitatory neurons: granule cells (marked by *Prox1*), CA1 pyramidal neurons (marked by *Fibcd1*) and CA3 pyramidal neurons (marked by *Nectin3* and *Sulf1*), as well as the five types of interneurons previously described in the mouse hippocampus (*12*) (fig. S2, R to Y).

Additionally, we identified three small neuronal clusters in close proximity to CA3 pyramidal neurons (marked by *Nectin3* but not by *Sulf1*) that represented mossy cells (marked by *Rgs12*), athorny (or deep) CA3 pyramidal neurons (marked by *Col6a1* and *St18*) (*13*–*15*) and a group of cells with a very distinct set of unique markers (*Glp1r*, *Ankrd22*, *Gpr6*, *Penk*, *Ecel1*, *Znf804b*, *Cux1*, *Fn1*) (fig. S2, Z to Ad). These likely represented the previously described Glucagon-like peptide-1 receptor (Glp1r) expressing cells (*16*), that are located in the hilus and are sometimes confused with mossy cells (*17*). The rest of the glutamatergic neurons formed several complex clusters and represented neurons from the two adjacent regions to the hippocampus: the subiculum and the entorhinal cortex. Finally, we used three independent confirmation strategies to validate our clustering. First, we performed a hierarchical classification of all the cell types identified in the golden hamster hippocampus and found related cell types clustered together in the dendrogram (Fig. 1D). Second, we compared our clusters with previously published clusters defined in mouse (*12*) and human (*18*) hippocampus and found a high degree of similarity (fig. S3A), indicating a high degree of evolutionary conservation. Third, we represented the UMAP with the uncorrected data (no integration with Harmony) and verified that the same cell types previously described clustered separately (fig. S3B).

### Transcriptional changes in the hippocampus across the hibernation cycle

We analyzed the changes that occurred during hibernation within our single-cell atlas (Fig. 2A) and verified that these changes are not driven by the integration pipeline (fig. S4A). Principal component analysis demonstrated that euthermic, torpor and arousal samples clustered together but clearly separated per condition (fig. S4B). The cellular composition of the hippocampus did not overtly change between euthermic, torpor and arousal, with only minor changes detected that may be either “method” or “biology”-associated (fig. S4C). However, profound transcriptional changes were seen across the hibernation cycle within each cell cluster (Fig. 2A). Differential abundance testing results of cell neighborhoods with Milo (*19*) revealed that neurons and glia behaved differently during hibernation (Fig. 2B). Neurons drastically changed their transcriptome during torpor, and in arousal they transitioned back to a cellular state that was highly similar to neurons in control. However, glial cells did not return to a control-like state in arousal and acquired an entirely novel transcriptional signature that was more closely related to torpor than to control. Quantification of the log-fold changes of enriched or depleted neighborhoods within different cell-types showed that microglia and astrocytes underwent the largest transcriptional shift across the hibernation cycle; they were the only cell types that always appeared in the top three when cross-comparing all different stages of hibernation (Fig. 2, C to E).

**Fig. 2.**
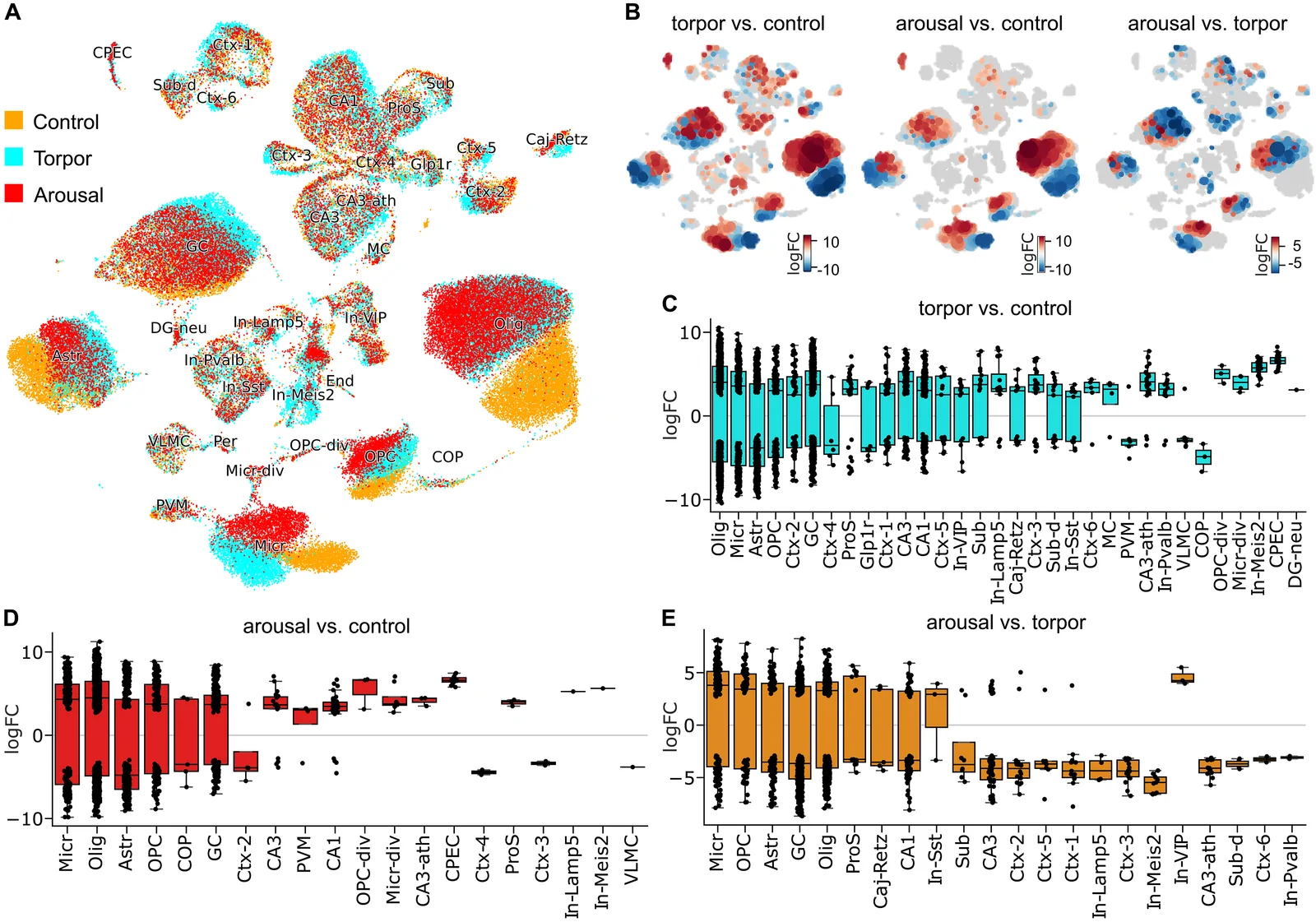
Hibernation-induced transcriptional changes. (**A**) UMAP plot depicting hippocampal cells from hamsters within different hibernation states, after batch integration with Harmony. (**B**) Differential abundance testing results of cell neighborhoods with Milo. Dot size represents size of neighborhood, blue indicates depletion and red enrichment across different hibernation state comparisons. (**C** to **E**) The log-fold changes of enriched or depleted neighborhoods with a spatial FDR < 0.05 within different cell-types are shown. The largest variance is apparent in glia, suggesting these are the cell-types with the largest cell state changes.

Differential gene expression analysis using DESeq2 (*20*) identified thousands of differentially expressed genes (DEGs) between control, torpor and arousal in all the three major glial and neuronal clusters (fig. S4D). Many of the torpor-associated DEGs in these six cell types (fig. S5A) belonged to five common gene ontology (GO) cellular component categories: “mitochondria,” “ribosome,” “organelle membrane,” “myelin sheath,” and “synaptic vesicle,” and three biological processes: “mitochondrial organization and transport,” “cellular respiration,” and “cytoplasmic translation” (fig. S5B). Mitochondrial genes that were highly upregulated in torpor included several complex I subunits (*Ndufa13, Ndufb5, Ndufs7*; Fig. 3A). These are likely required for the metabolic transition to torpor, in which ATP levels in the brain are maintained equal to control despite the drastic decrease in caloric intake and blood flow (*21*). Interestingly, ribosomal genes (*Rpl36*, *Rplp1, Rps23*; Fig. 3A) were also upregulated in torpor despite the dramatic decrease in protein translation reported in the brain of torpid animals (*22*). We validated that the levels of several ribosomal proteins were similarly increased during torpor (Fig. 3, B to D, and fig. S6), suggesting that the upregulation of ribosomal genes and proteins in torpor prepares the transition to arousal in which multiple mRNAs need to be rapidly translated.

**Fig. 3.**
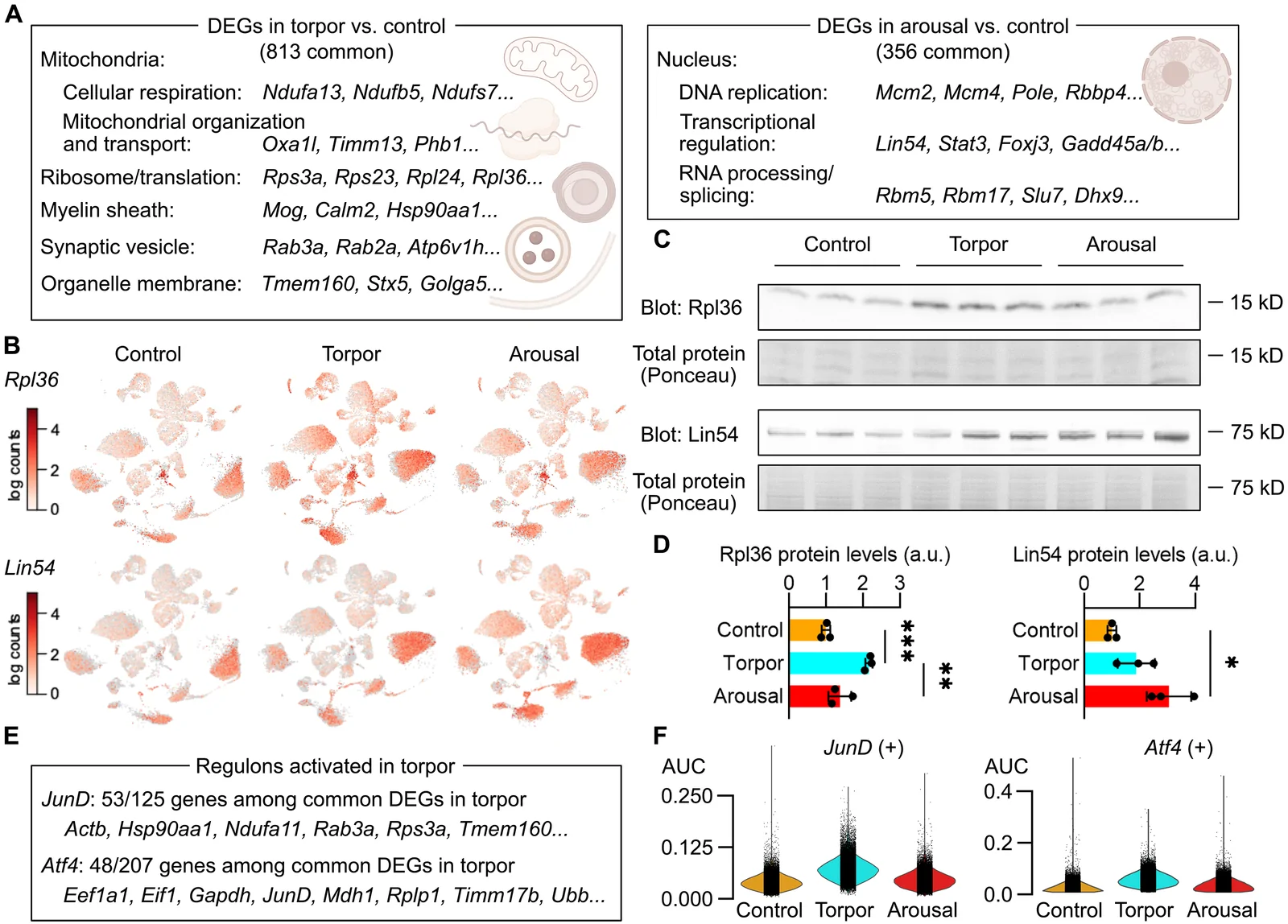
Common hibernation-induced genes and regulons. (**A**) Summary of the differentially expressed genes (DEGs) common to the main six hippocampal cell types (astrocytes, microglia, oligodendrocytes, granule cells, CA1 and CA3 pyramidal neurons). Cartoons Created in BioRender. Verstreken, P. (2026) https://BioRender.com/heg9hu0, https://BioRender.com/dn0zs8f, https://BioRender.com/m7nzbpo, https://BioRender.com/19fgw73, https://BioRender.com/asbnd2u. (**B**) UMAP plots showing the expression of ribosomal gene Rpl36 and transcription factor Lin54 across the hibernation cycle. (**C**) Western blots showing Rpl36 and Lin54 protein levels in hippocampal homogenates from control, torpor and arousal hamsters. (**D**) Quantification of C. Graphs depict mean ± SD (*P < 0.05; **P < 0.01). Detailed statistical results are provided in table S1. (**E**) Summary of the transcriptional networks (regulons) activated in torpor across different cell types, and example of DEGs included in those regulons. (**F**) Violin plots showing the expression of all genes included in the JunD and Atf4 regulons in control, torpor and arousal.

In respect to torpor-induced changes in neurons, over 3,000 genes are commonly differentially expressed across the three major hippocampal neuronal subtypes (granule cells and CA1 and CA3 pyramidal neurons). Among these genes, *Rab3a* is the most upregulated, together with other small GTPases involved in vesicle trafficking and membrane reorganization, such as *Rab2a*, *Arf1*, and *Arf5* (fig. S5A). Additional highly upregulated torpor genes include chaperones such as *Hsp90ab1* and *Hsp90aa1*, implicated in protein stability and degradation in response to stress, and stathmins (*Stmn2*, *Stmn3* and *Stmn4*), which regulate cytoskeletal dynamics (fig. S5A). Notably, the five interneuron classes showed a similar upregulation of these genes during torpor; this suggests that together with ribosomal and mitochondrial changes, they constitute a core neuronal adaptive response to cold and nutrient deprivation. Neuronal type-specific GO terms enriched in torpor include “axon” and “actin cytoskeleton” in CA3 pyramidal neurons, “neurotransmitter receptor activity” and “neuron projection guidance” in CA1 pyramidal neurons (fig. S5B). Some of these molecular adaptations seem to support a degree of synaptic remodeling as previously observed in the golden hamster hippocampus (*23*, *24*), but further studies will be required to address this in more detail.

Analysis of DEGs in arousal was more complex: in contrast to torpor, different cells tended to upregulate different genes in arousal (fig. S7A). Thus, no common cellular components or biological functions were observed when GO term analysis was performed (fig. S7B). Contrarily, arousal-associated DEGs belonged to different GO terms: “DNA replication” was enriched in microglia and, surprisingly, in pyramidal neurons, whereas “circadian rhythm” was enriched in microglia and astrocytes (it was also enriched in torpor only in astrocytes). “Autophagy” was enriched only in astrocytes, whereas “RNA processing and splicing” was enriched only in microglia. Nevertheless, several nuclear genes were upregulated in arousal in both neurons and glia (Fig. 3A) including *Lin54*, a transcription factor which is involved in cell cycle control and that is also upregulated at the protein level (Fig. 3, B to D). In contrast, *Fos*, another transcription factor which is commonly used as a marker of cellular activity, was upregulated in arousal, but only in glia (fig. S7A).

To further investigate hibernation-associated changes in transcription factor regulatory networks (regulons) we used SCENIC (*25*), which enabled to define differentially expressed regulons throughout the hibernation cycle. The majority of the changes in regulon expression were uniformly present across different cell types and most of them occurred in torpor. Some of these changes were maintained in arousal, whereas others reversed back to control levels. For instance, we found that *JunD*, an antiapoptotic transcriptional network, and *Atf4*, which regulates the integrated stress response, were strongly activated during torpor and returned to control levels in arousal (Fig. 3, E and F). While none of the regulons were specifically up/downregulated in arousal, other regulons such as *Esrra*, a key coordinator of energy metabolism and autophagy, and *Tcf3*, which represses neuronal differentiation, were significantly reduced in torpor and arousal compared to controls (data not shown). Together with the analysis of DEGs and GO terms, these data reveal the complex regulatory programs that impact hibernation cell state changes.

### Hibernation-induced changes in astrocytes

Astrocytes perform essential functions in the brain such as neuronal support and brain-blood barrier maintenance. We found large transcriptome changes in astrocytes across the hibernation cycle, with torpor astrocytes clearly separated from control ones in the UMAP and arousal astrocytes in between the other two subtypes (Fig. 2A). Apart from mitochondrial and ribosomal genes, DEGs in torpor included *Tigit*, a receptor involved in the immune response, *Hpse2 and Timp1,* which are implicated in extracellular matrix remodeling, and *Letm1*, a mitochondrial regulator of ion transport (fig. S5A). Interestingly, *Letm1* controls ionic and molecular clock rhythms (*26*) and it was specifically upregulated in astrocytes in torpor and arousal. Other clock genes such as *Cry1*, *Cry2*, and *Bmal1* (*27*) were also differentially expressed in astrocytes, especially in arousal (fig. S5A), and GO terms related to circadian rhythm and photoperiodism were highly enriched in astrocytes in both torpor and arousal (figs. S5B and S7B). Hence, astrocytes appear to connect hibernation to immune response and circadian rhythmicity.

One of the most differentially regulated genes within astrocytes was the transcription factor *Fos* which is a general marker for active astrocytes (*28*) and was significantly increased in arousal compared to torpor (and control). We co-labelled hamster hippocampi with c-Fos and Gfap (a marker for activated astrocytes) antibodies and found a robust increase in the percentage of c-Fos-Gfap-positive astrocytes in arousal compared to control and torpor (Fig. 4, A and B). Labeling coronal brain sections with Gfap also showed that astrocytes in euthermic hamsters largely appeared in two hippocampal regions: the dentate gyrus and the stratum lacunosum moleculare (fig. S8A). In contrast, CA3 and CA1 regions contained only a small number of labeled astrocytes and these were mostly surrounding blood vessels as part of the brain-blood barrier (fig. S8A). These observations contrasted with mouse astrocytes, which located uniformly across the entire hippocampus (fig. S8A). Interestingly, during hibernation the location of Gfap-positive astrocytes changed profoundly: in torpid animals, the percentage of labeled astrocytes within the dentate gyrus was reduced and the percentage of labeled astrocytes within the CA3 and CA1 regions was increased, whereas in arousal an intermediate distribution was observed (Fig. 4, C and D, and fig. S8B).

**Fig. 4.**
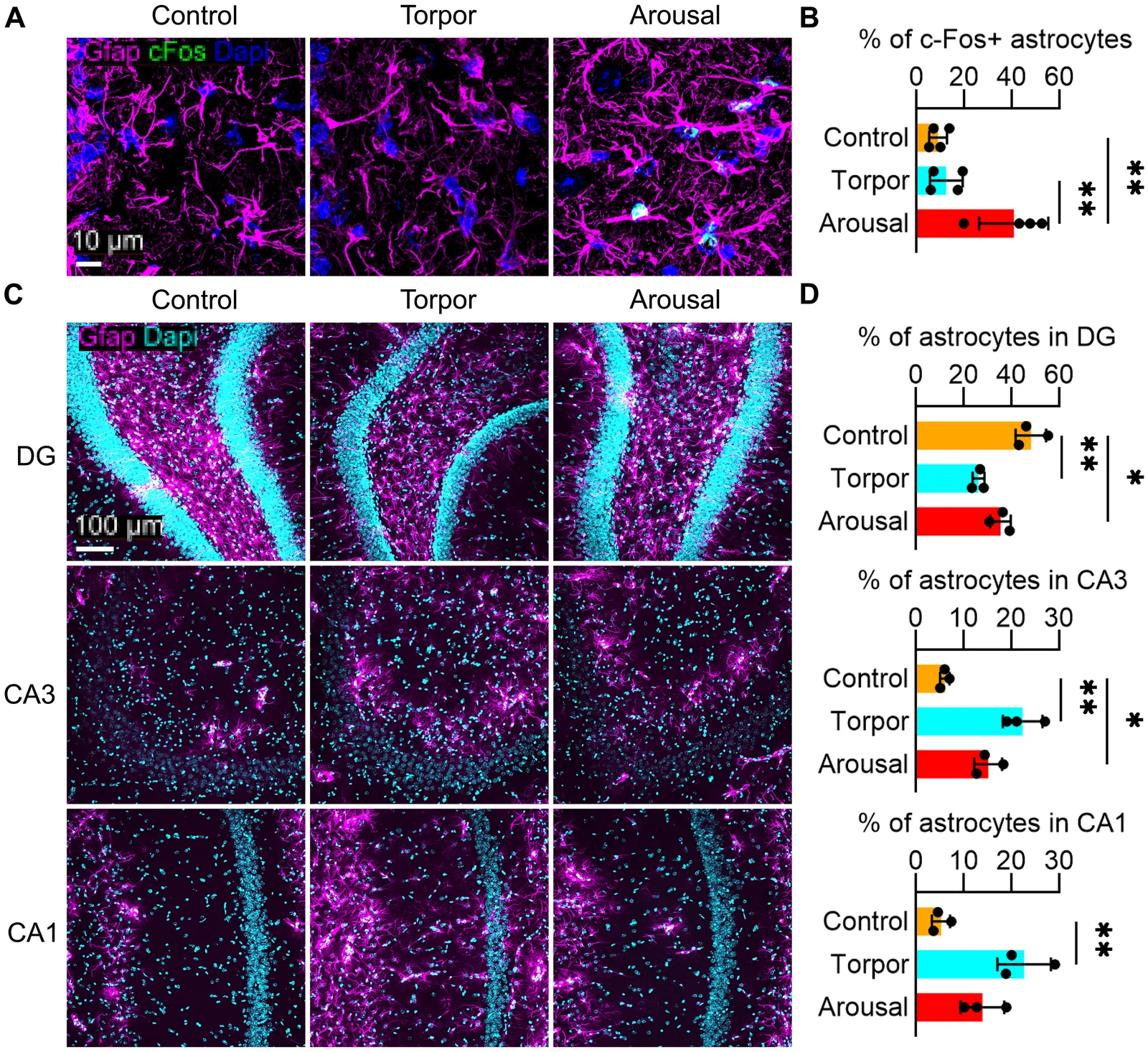
Hibernation-induced changes in astrocytes. (**A**) Representative confocal images of astrocytes from control, torpor and arousal hamsters labeled with astrocytic marker Gfap, cellular activity marker c-Fos and nuclear marker Dapi. (**B**) Quantification of (A). Graph depicts mean ± SD for *N* = 4 different animals (**P < 0.01). Detailed statistical results are provided in table S1. (**C**) Representative confocal images of 3 different hippocampal regions (DG, dentate gyrus) from control, torpor and arousal hamsters labeled with the astrocytic marker Gfap and nuclear marker Dapi. (**D**) Quantification of (C). Graphs depict mean ± SD (*P < 0.05, **P < 0.01). Detailed statistical results are provided in table S1.

To further investigate the time dynamics of the astrocyte changes in arousal, we also labelled astrocytes from hamsters 24 hours after the onset of arousal (arousal-long) as well as animals that naturally escaped from several cycles of hibernation and remained euthermic for at least one week without re-entering in torpor (post-hibernation). We found that even after 24 hours of arousal, astrocytes had not yet regained the spatial distribution characteristic of control animals. In contrast, this pattern was fully restored in the post-hibernation condition (fig. S8, C and D). These findings indicate that astrocytes in arousal remain in a distinct physiological state, rather than simply returning to the euthermic baseline. Moreover, whereas neurons rapidly revert their transcriptional profile in the first hours after torpor, astrocytes appear to maintain a transient “activated” state even 24 hours after arousal. Together, our data show that hibernation induces a differential activation of hippocampal astrocytes that seems to be partially maintained during arousal bouts.

### Microglial states in hibernation, Alzheimer’s disease, aging and brain injury

Microglia are the main immune cells in the brain and there is enormous morphological and transcriptional plasticity in response to different adverse conditions such as those in AD (*29*). Microglia were among the most affected cell types in our hibernation cell atlas; in contrast to other cell types, microglia from different hibernation stages clustered separately within the UMAP (Fig. 2A). Thus, we performed unsupervised subclustering and found that microglia from control (“Micro-C”), torpor (“Micro-T”) and arousal (“Micro-A”) animals grouped in three distinct subclusters, with a fourth subcluster containing dividing microglia (“Micro-D”) (Fig. 5A). “Micro-D” was mostly composed (67%) of cells from aroused animals (Fig. 5B) and genes involved in cell cycle progression and cell division (*Ezh2*, *Brip1*, *Mcm5*) were upregulated in “Micro-A,” together with pro-inflammatory genes (*Cd83*, *Nfkb1*) and cytokines (*Ccl3*, *Il1b*) (Fig. 5C and fig. S9A). Conversely, “Micro-C” was characterized by high expression of homeostatic genes such as *Cx3cr1* and *P2ry12*, whereas “Micro-T” expressed ribosomal (*Rps3a*, *Rpl13*) and iron storage genes (*Fth1*, *Ftl*) (Fig. 5C and fig. S9A). We used RNAScope to validate the presence of three distinct microglial populations in control, torpor and arousal animals marked by *Cx3xr1*, *Rps3a*, and *Cd83*, respectively (fig. S9, B and C).

**Fig. 5.**
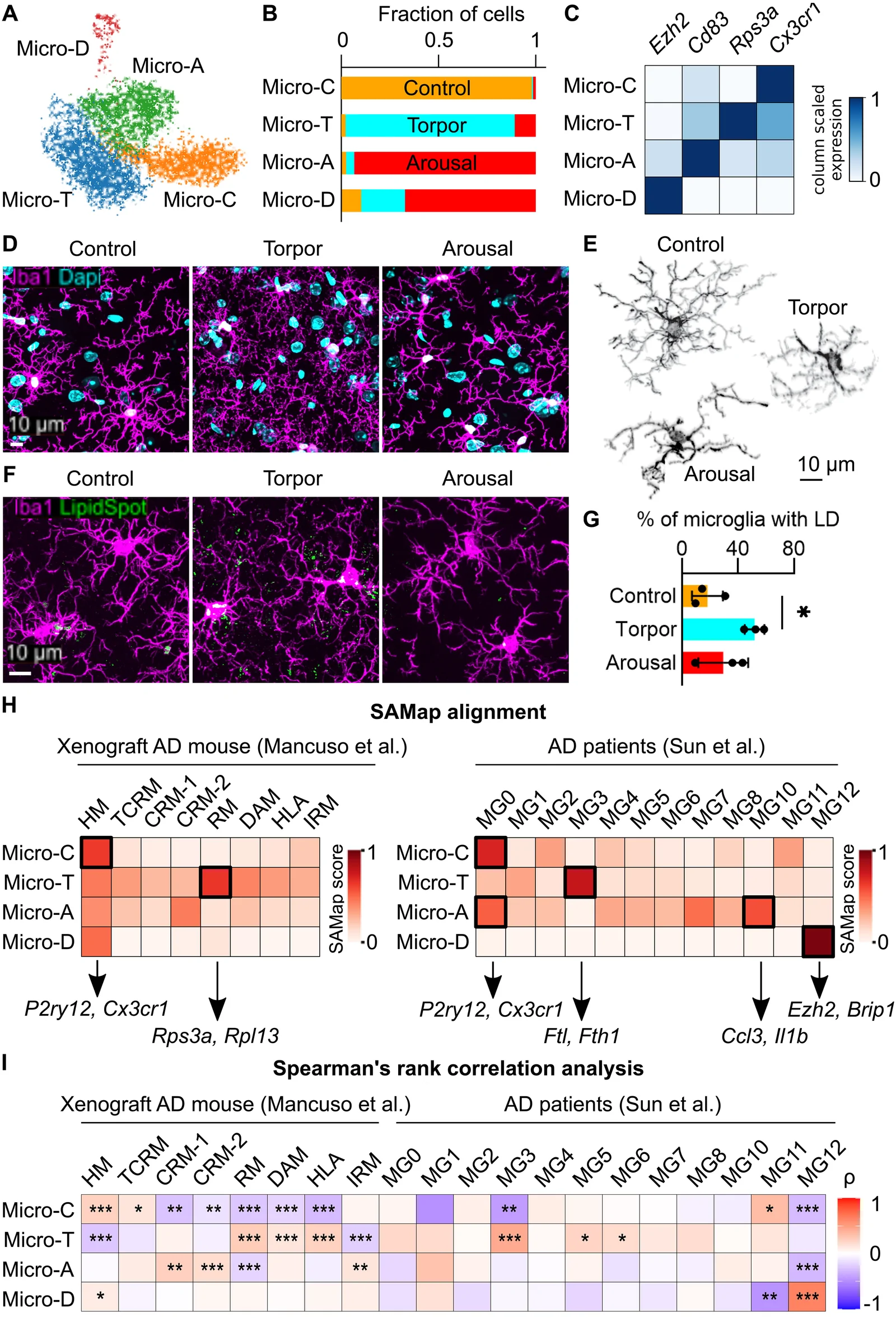
Microglial states in hibernation and Alzheimer’s Disease. (**A**) Unbiased Leiden clustering with resolution 2.0 identifies four subclusters of hamster microglia, termed “Micro-C” (control), “Micro-T” (torpor), “Micro-A” (arousal), and “Micro-D” (dividing). (**B**) Fraction of cells from control, torpor and arousal animals for each of the four microglial subclusters. (**C**) Column scaled expression of *Ezh2*, *Cd83*, *Rps3a*, and *Cx3cr1* in the different subtypes of hamster microglia. (**D**) Representative confocal images of microglia from control, torpor and arousal hamsters labeled with microglial marker Iba1 and nuclear marker Dapi. (**E**) Segmented images of individual microglia from control, torpor and arousal hamsters extracted with Napari. (**F**) Representative confocal images of microglia from control, torpor and arousal hamsters labeled with microglial marker Iba1 and lipid droplet marker LipidSpot. (**G**) Quantification of (F). Graph depicts mean ± SD (*P < 0.05). Detailed statistical results are provided in table S1. (**H**) Graphs depicting the SAMap alignment scores of the four subtypes of hamster microglia with the different microglial states defined in a xenograft mouse AD model and in AD patients. Alignments with a SAMap score higher than 0.5 and example marker genes are highlighted. (**I**) Table depicting Spearman’s rank correlation coefficient (ρ) for the four subtypes of hamster microglia with the different microglial states defined in a xenograft mouse AD model and in AD patients. Statistical significance was determined using two-sided test against the null hypothesis of ρ = 0.

Iron-dependent accumulation of polyunsaturated fatty acids into lipid droplets is associated with ferroptosis and other ferroptosis-related genes, such as *Gpx4* and *Acsl1,* are also dysregulated in microglia during hibernation (fig. S9A). We thus aimed to detect lipid droplets in hamster microglia. We first labelled coronal brain sections with microglial marker Iba1. Microglia uniformly populated the hamster hippocampus and hibernation did not induce changes in their location (fig. S9D). However, the morphology of microglial cells drastically changed: in torpid hamsters (mostly “Micro-T”) microglial processes were shorter and thinner compared to euthermic animals (“Micro-C”) and microglia from hamsters in arousal (“Micro-A”) showed an intermediate phenotype (Fig. 5, D and E), in line with earlier findings (*30*, *31*). We then labelled hamster hippocampi with LipidSpot that stains lipid droplets and observed that a significantly larger number of microglia from torpid hamsters contained lipid droplets compared to controls (Fig. 5, F and G). This phenotype was partially reversed in microglia from aroused animals (Fig. 5, F and G). Thus, hibernation induces morphological changes and the accumulation of lipid droplets in microglia.

Lipid droplet accumulation in microglial cells represents a proinflammatory state that is also associated with aging (*32*). This phenotype is furthermore linked to the different ApoE risk variants and it correlates with the progression of cognitive decline in AD (*33*). Hence, we used SAMap alignment (Fig. 5H) and Spearman’s rank correlation analysis (Fig. 5I) to compare hibernating hamster microglia with the different human microglial states described in AD patients (*34*) and a xenotransplantation animal model where human microglia are exposed to amyloid pathology in vivo (*35*). The homeostatic microglia cell state (HM) found in this xenotransplantation model best matched the hamster “Micro-C” cluster and negatively correlated with most activated microglia states. “Micro-T” cells most closely related to the ribosomal response (RM) state and positively correlated as well with disease-associated response (DAM) and antigen-presenting response (HLA). Interestingly, these two responses were associated to Aβ pathology in the xenograft AD model, whereas Aβ plaques were not detectable in the brain of hibernating (or control) animals (fig. S10A). However, as previously described, AT8-positive p-Tau accumulated in the hippocampus of hamsters in torpor and arousal (fig. S10, B to D). “Micro-A” mapped best to cytokine responses (CRM-1 and -2) and interferon response (IRM). Hence, the microglial response in hibernation bears strong similarity to the neuroinflammatory states in a xenograft AD mouse model, and it is induced independent of Aβ.

We therefore compared microglia from hibernating animals to those found in AD patients (*34*). “Micro-D” matched well to human cycling microglia (MG12) and “Micro-C” strongly mapped to human homeostatic microglia (MG0). The best match for “Micro-T” was MG3, characterized by the expression of ribosome biogenesis (*Rps11, Rpl32*) and iron storage genes (*Ftl, Fth1*). Interestingly, “Micro-T” also positively correlated with states MG5 (phagocytic) and MG6 (stress-response). These associations are consistent with a coordinated stress-response program induced by cold and nutrient deprivation. “Micro-A” state matched with two human states: homeostatic (MG0) and inflammatory state III microglia (MG10). MG10 shows the greatest response in early stages of AD and MG10 and “Micro-A” were both enriched for cytokine genes such as *Ccl3* and *Il1b*. In contrast, microglial inflammatory states I (MG2) and II (MG8), which are enriched for other inflammatory markers (*Cpeb4, Tmem163, Lrrk2, Spon1*) and are responsive at late stages of AD, did not match well with “Micro-T” or “Micro-A” states. Additionally, to validate our results we performed gene set enrichment analysis (fig. S11A) and module scoring (fig. S11B), which further confirmed the strong association of hibernation-induced microglial response and AD-related microglial states.

Finally, to assess to what extent the similarities between hibernation and AD microglial responses are specific, we compared hamster microglia to mouse microglia from aging and brain injury models (*36*). In this case, Spearman’s rank correlation and gene set enrichment analysis did not find any significant associations. However, evaluating the expression of the markers associated to these states in hamster microglia revealed strong upregulation of specific gene modules in “Micro-T” and “Micro-A.” The majority of genes upregulated in aged microglia were enriched in “Micro-T”; this list includes iron storage genes *Fth1* and *Ftl*, chaperone *Hsp90ab1* and AD-associated gene *ApoE* (fig. S12A). In addition, a smaller group of genes upregulated in aged microglia was enriched in “Micro-A,” including inflammatory markers such as *Il1b*, *Ccl3*, and *Cd83* (fig. S12A). Similarly, most of the genes upregulated in response to brain injury were enriched in “Micro-T,” including ribosomal genes and again *Fth1*, *Ftl*, and *ApoE* (fig. S12B). However, it is important to note that a large fraction of the genes downregulated in response to brain injury were also enriched in “Micro-T,” including antiapoptotic transcription factor *JunD* (fig. S12C). These genes potentially represent hibernation-specific protective responses that prevent neuronal degeneration and preserve brain function in the context of a neuroinflammatory surrounding. Again, we performed module scoring analysis and further confirmed these findings (fig. S12D). In summary, the microglial response in hibernation bears strong similarity to microglial inflammatory responses in AD and, to a lesser extent, in brain injury and aging.

## DISCUSSION

In this study, we present the first single-cell hibernation atlas, representing a groundbreaking advance and providing a valuable resource for the research community. We identify 34 different cell clusters within the golden hamster hippocampus, including the most common cell types that show a high degree of similarity with their mouse and human counterparts. Additionally, we identify rare neuronal populations such as athorny CA3 pyramidal neurons and Glp1r-expressing neurons that have been difficult to resolve in other single-cell atlases. Moreover, our dataset includes samples from animals in the two different phases of the hibernation cycle, torpor and arousal, and control euthermic animals. Hibernation requires fast and reversible changes in the brain, and here we uncover the cell type-specific transcriptional programs associated with these adaptations. We observe that hibernation induces common transcriptional responses in all different cell types, demonstrating that the transitions between different hibernation phases are regulated by coordinated changes in gene expression and gene regulatory networks. Our data can be used to understand previously observed (but mechanistically unexplained) phenotypes. For instance, we observe an increase of mitochondrial complex I genes in torpor, which could explain the metabolic shift that occurs in this state (*21*) and which is a typical compensatory mechanism observed in response to cellular stress and neurodegenerative diseases (*37*, *38*).

In parallel, we observe notable cell-type-specific responses, with microglia showing the most pronounced transcriptional changes. Hibernation triggers an inflammatory-like state in microglia marked by specific gene expression signatures. Interestingly, these states closely resemble some of the neuroinflammatory states described in AD patients and animal models. Although neuroinflammation is often regarded as detrimental in AD (*39*), increasing evidence points to potential protective functions of activated microglia in early disease stages (*40*, *41*). In hibernation, inflammatory-like states in microglia closely resemble AD-associated responses but they do not cause lasting brain damage (*24*). In particular, microglial activation during hibernation mirrors ribosomal and early pro-inflammatory states associated with AD, suggesting that these states might represent a conserved protective and reversible response to different types of stress (e.g., lack of nutrients and cold like in hibernation, or the accumulation of toxic proteins like in disease). Importantly, the neuroinflammatory states typical of late-stage AD are not observed during hibernation, suggesting that these may be associated to an irreversible and damaging response. Further research will be needed to confirm this hypothesis and determine whether hibernation can serve as a model to dissociate protective from detrimental neuroinflammatory responses relevant to AD.

Microglial ribosomal response has been previously characterized as an activated microglial state and is defined by the expression of ribosomal genes as well as oxidative stress and iron storage genes (*Ftl*, *Fth1*). Here, we observe that the torpor-induced microglial state resembles the ribosomal state described in human microglia from an AD xenotransplantation model and patients (*34*, *35*). Interestingly, we also observe that this cell state is linked to the accumulation of lipid droplets inside microglia, which strongly correlates with aging and AD progression but had not been associated to a specific microglial activation state before. The fact that torpid microglia show accumulation of lipid droplets suggests that in AD this phenotype is induced by the ribosomal activation state and might be part of a protective response against oxidative stress. On the other hand, the arousal-induced microglial state resembles a type of cytokine response (marked by *Ccl3* and *Il1b*) that is associated with early stages of AD, although with a lower degree of similarity. However, the alignment scores were considerably higher than for the other pro-inflammatory states linked to late AD stages. In addition, several markers associated to common hibernation and AD microglial states (including ferritin, cytokine/interleukin and ribosomal genes) are similarly upregulated in aging and brain injury, suggesting that they constitute a common adaptive microglial response conserved across species.

Neuroinflammation is a heterogenous response associated with multiple brain diseases and it is often difficult to separate pathological processes from endogenous protective mechanisms. Previous studies have identified multiple distinct microglial transcriptional states that are associated to different stages of aging and AD (*34*, *35*). However, the diversity of cell states and responses confounds the design of therapeutic strategies to suppress neuroinflammation, which proved to be ineffective to prevent or delay cognitive decline in AD patients (*39*). Our results suggest that hibernation could provide a unique physiological model to investigate the differences between adaptive, reversible, and potentially protective inflammatory states and those associated with progressive neurodegeneration. By systematically defining the shared and cell-type-specific transcriptional responses during the hibernation cycle, our work disentangles physiological neuroinflammatory mechanisms from those driven by disease pathology. This provides the necessary reference point for interpreting neuroinflammatory signatures in disease and could help identifying the responses that should be potentiated or inhibited to prevent or revert disease progression.

## MATERIALS AND METHODS

### Induction of hibernation

Three-month-old male golden hamsters were ordered from Janvier labs. Euthermic (control) hamsters were maintained on a diurnal 12-hour light/dark cycle and 21°C. For the induction of hibernation, after two weeks of adaptation to the animal facility, hamsters were transferred to a hibernaculum, in which they were housed in individual cages equipped with movement detectors. In a two-week period, temperature was progressively lowered from 21°C to 4°C and light intensity and daytime were progressively reduced to reach complete darkness. Analysis of the movement recordings was used to define when the hamsters enter “torpor” and “arousal” phases (see Fig. 1, A and B). Typically, after one to two months the majority of the hamsters placed in the hibernaculum entered into the hibernation cycle, consisting of torpor phases of no movement recorded (with an average duration of 4–5 days) and arousal movement bouts (with an average duration of 1–2 days). Hamsters were euthanized within the initial hours of the torpor and arousal phases, and after at least one full cycle of torpor and arousal. All experiments involving the use of animals were conducted according to the KU Leuven ethical guidelines and approved by the KU Leuven Ethical Committee for Animal Experimentation (ECD: P053/2021).

### Single nucleus sample preparation

Animals were anaesthetized with an intraperitoneal injection of pentobarbital and decapitated. Hippocampi were dissected out of the brain and immediately frozen in liquid nitrogen. Hippocampi were thawed in ice-cold homogenization buffer (containing 10 mM Tris pH 7.5, 191 mM NaCl, 1 mM CaCl_2_, 21 mM MgCl_2_, 25 mM KCl, 250 mM sucrose, 1 mM β-mercaptoethanol, 0.03% Tween-20, 0.01% bovine serum albumin, cOmplete Protease Inhibitor Cocktail from Roche and RNasin Plus RNase Inhibitor from Promega) and homogenized by 10–15 gentle strokes with glass pestles. Hippocampal homogenates were filtered through a 70 μm cell strainer and centrifuged at 500x g for 5 minutes. Pellets were resuspended in 2.65 mL of homogenization buffer (without Tween-20) and added to the same volume of gradient medium (50% OptiPrep Density Gradient Medium from Merck containing 10 mM Tris pH 7.5, 1 mM CaCl_2_, 5 mM MgCl_2_, 75 mM sucrose, 1 mM β-mercaptoethanol, cOmplete Protease Inhibitor Cocktail and RNasin Plus RNase Inhibitor). Samples were layered on top a 29% Cushion solution (29% OptiPrep Density Gradient Medium containing 60 mM KCl, 24 mM Tris pH 8, 12 mM MgCl_2_, 100 mM sucrose) and centrifuged in a SW41Ti rotor (Beckman Coulter) at 7,700 rpm and 4°C for 30 minutes. Supernatant was discarded and the nuclei pellet at the bottom of the centrifugation tube was resuspended in PBS containing 2% bovine serum albumin and RNasin Plus RNase Inhibitor and filtered through a 40 μm Flowmi cell strainer.

### cDNA library preparation and sequencing

Library preparation for the single cell RNA sequencing was performed using 10X Genomics Chromium Single Cell 3’ Kit, v3.1 NextGEM chemistry (10X Genomics, Pleasanton, CA, USA). The nuclei count and concentrations were calculated using LUNA dual florescence cell counter (Logos Biosystems) and a targeted cell recovery of 10,000 cells was aimed for each of the samples. After cell count and quality control, samples were immediately loaded onto the Chromium Controller. Single cell RNAseq libraries were prepared using manufacturers recommendations (Single cell 3′ reagent kits v3.1 user guide; CG000204 Rev. D), and at the different check points the library quality was assessed using Qubit (ThermoFisher) and Bioanalyzer (Agilent). With a sequencing coverage targeted for 50,000 reads per cell, single cell libraries were sequenced either on Illumina’s NovaSeq 6000 platform using single-end sequencing workflow as recommended by 10X Genomics Workflow; v3.1 read parameters (28–8–0-91 cycles), or using BGI’s MGISEQ-2000 sequencing platform. Sequencing of the10X libraries using the MGISEQ-2000 sequencing platform was performed at the MGI Hong Kong SAR sequencing facility. Before being able to sequence on the MGI platforms, the 10X libraries underwent a conversion step using MGIEasy Universal DNA Library Prep kit. Briefly, the final 10X sequencing libraries were circularized using the splint-ligation step and the circularized libraries were converted to single stranded DNA copies. DNA nanoballs were prepared from the circularized ssDNA using Rolling Circle Amplification (RCA). Generated DNA libraries were then flown through the patterned flow cell of the MGISEQ-2000RS High-Throughput Sequencing kit. For a targeted sequencing saturation of 50–60%, sequencing was performed at a depth of 30,000–60,000 reads per cell. Single-end sequencing was performed with reads of 28 bps (read 1), 100 bps (read 2) and 10 bps (index 1).

### Generation of an updated *Mesocricetus auratus* genome index

The *M. auratus* genome and annotation (Version 103) were downloaded from NCBI (GCF_017639785.1). Since the release of this genome annotation, further gene names have been defined by NCBI; to include these in our analyses, we downloaded the following file https://ftp.ncbi.nlm.nih.gov/gene/DATA/GENE_INFO/Mammalia/All_Mammalia.gene_info.gz (Downloaded on 3rd May 2023) which contains the latest gene names for all mammalian genomes. We updated each gene within the annotation GTF file based on their NCBI ID with the latest gene name. Finally, genes present on the mitochondrial genome were prepended with the prefix “MT-”. This GTF file was filtered with cellranger mkgtf with the following parameters:

--attribute = gene_biotype:protein_coding

--attribute = gene_biotype:lincRNA

--attribute = gene_biotype:antisense

--attribute = gene_biotype:IG_LV_gene

--attribute = gene_biotype:IG_V_gene

--attribute = gene_biotype:IG_V_pseudogene

--attribute = gene_biotype:IG_D_gene

--attribute = gene_biotype:IG_J_gene

--attribute = gene_biotype:IG_J_pseudogene

--attribute = gene_biotype:IG_C_gene

--attribute = gene_biotype:IG_C_pseudogene

--attribute = gene_biotype:TR_V_gene

--attribute = gene_biotype:TR_V_pseudogene

--attribute = gene_biotype:TR_D_gene

--attribute = gene_biotype:TR_J_gene

--attribute = gene_biotype:TR_J_pseudogene

--attribute = gene_biotype:TR_C_gene

The final GTF and fasta files were then used to create a Cellranger index using Cellranger v6.0.2.

### Quality control and processing of single nuclei data

Raw BCL files were demultiplexed using bcl2fastq2 (v2.20.0) into fastq files, and these were aligned to the hamster genome created above and quantified using Cellranger (v6.0.2). Filtered count matrices output were loaded into Python 3.8.10 using Scanpy (*42*) (v1.9.1). First, predicted doublets were removed from the data using scrublet. To determine the threshold for filtering, we calculated the predicted number of doublets per sample using the formula: n_cells * ((n_cells / 1000) * 0.08). The cells were ordered by their scores, and the score of the cell at position predcited_num_doublets +1 was used as the threshold for filtering. After doublet removal, all samples were combined into a single object for further processing. Cells were further filtered by removing cells with more than 5% mitochondrial reads, less than 250 genes expression or more than 100,000 UMIs detected. Counts were next normalized to a total of 10,000 counts per cell and then log-normalized, highly variable genes were identified (min_mean = 0.0125, max_mean = 3, min_disp = 0.5), the total number of counts and % mitochondrial reads were regressed out and counts were scaled to unit variance with a zero mean and a max value of 10. A principal component analysis was performed, and the top 57 components were selected to use for downstream analyses.

### Clustering and cell-type analysis

Harmony (*43*) was next used to perform a batch effect correction using the date of collection of the samples as the batch category. These corrected PCA embeddings were then used for the calculation of the UMAP and t-SNE. Cluster detection was performed at multiple resolutions (0.4, 0.8, 1.0, 2.0, 4.0, 8.0) using the Leiden algorithm and the rank_genes_groups function was used to identify marker genes at each resolution. Leiden 2.0 was chosen as the base resolution for cell-type annotation. Therefore, established marker genes as well as cell-type mappings to mouse (*12*) and human single-nucleus (*18*) with SAMap (*44*) (see below) were used. Cluster 15 was split further at resolution 4.0 to separate CA1 from the prosubiculum, which are separated by a *Nts* expression gradient (*45*). Cluster 49 was further divided at resolution 4.0 to yield distinct endothelial and pericyte clusters.

### Differential expression analysis

Differential gene expression (DEG) testing was performed in a cell-type-specific pseudobulk manner, in order to better control for false discoveries due to pseudoreplication if each cell is treated as a unique repeat (*46*–*48*). Therefore, all UMIs of a given gene in all cells of a given cell-type, in a given condition and repeat were sum aggregated, which resulted in 3–4 pseudobulk “samples” per condition and cell-type. Subsequently, DESeq2 (*20*) was used to fit negative binomial models, comparing torpor vs. control, arousal vs. control and arousal vs. torpor while regressing out batch effects with the design formula ∼experimental_date+hamster_condition. Finally, the Wald test was used for differential gene expression testing and log2 fold changes were then shrunk with type = ‘apeglm’ (*49*). *P* values were corrected with the Benjamini-Hochberg method with <0.05 as a threshold. Gene set enrichment analysis of gene ontology (GO) terms was carried out with the clusterProfiler (v 3.18.1) (*50*) gseGO() function and DEGs sorted according to log2-fold changes. To reduce the complexity of the resulting plots, GO terms were grouped based on similarity with the Wang method (*51*) in the mgoSim() function of the GOSemSim (*50*) R package (v2.16.1) and the reduceSimMatrix() functions in rrvgo (v1.2.0) (*52*) as implemented in the go_reduce() wrapper (*53*).

### Mapping of mouse and human datasets

To perform cross-species alignment of hamster clusters with previously established cell-types in mouse and human single-nuclei datasets, we used the self-assembling manifold mapping algorithm (SAMap) (*44*). For the purpose of testing cross-species cell-type homologies, we made use of a whole-mouse brain single-nucleus dataset encompassing hippocampus annotations (*12*) as well as a human hippocampus and entorhinal cortex dataset (*18*). To map healthy human and disease-associated microglia cell-states, we used one dataset containing microglia from healthy and Alzheimer’s disease (AD) human brains (*34*) as well as a dataset that encompasses human microglia transplanted in control and AD mouse models (*35*). To this end, count matrices were downloaded and used with the provided cell-type annotations in the metadata. SAMap v1.0.15 was run according to the author recommendations [https://github.com/atarashansky/SAMap/blob/main/SAMap_vignette.ipynb]. Briefly, in an initial step, reciprocal orthologs between hamster and mouse/human were extracted with the map_genes.sh script (*44*) and BLAST+ 2.11.0 with the coding sequences of the updated *M. auratus* genome and annotation (Version 103, see above), GRCm38 and GRCh37. Only the top transcript for each gene was retained to then build a joint, lower-dimensional manifold representation with the SAMap run() function. After stitching together hamster-human and hamster-mouse cells from the different datasets, alignment scores were calculated with the get_mapping_scores() function to be able to evaluate cross-species cell-type similarities.

### Compositional changes

Cluster-free differential abundance testing was carried out with the pertpy (v0.4.0) implementation of Milo according to the authors recommendations (*19*). Therefore, in a first step the neighborhood graph was calculated based on our harmony integrated dataset with the scanpy preprocessing.neighbors() function, the ‘umap’ method and the number of neighbors set to 33. The number of all cells in each neighborhood per condition was then calculated and subsequently differential abundance was calculated with the Milo implementation of edgeR’s quasi-likelihood F-test. We used the design formula ∼date+condition to remove batch effects and spatial FDR correction was applied to the resulting P-values (*54*). Neighborhoods were assigned to hamster cell-types if >75% of cells were of the same identity. This allowed us to rank cell-types according to their variance of significantly deregulated neighborhoods. Cell-type based differential abundance testing across conditions were carried out with scCODA (as part of pertpy v0.4.0), which uses a Bayesian model that accounts for the compositionality of the multi-condition single-cell datasets to better control for false discoveries (*55*). Best practices as outlined in https://www.sc-best-practices.org/conditions/compositional.html were followed. CA1 was selected as a reference cell-type and experimental date and condition included as covariates to correct for batch effects. We report credible effects below the indicated FDR. Similar results were obtained with the automatic detection of a reference cell-type – where PVM was identified – and solely using condition as a covariate.

### Transcriptional network analysis (SCENIC)

Identification of transcriptional networks was performed using pySCENIC (v0.11.2 + 19.gfaa0216). First, a SCENIC database for hamster was created utilizing the tools available at the following GitHub repository: https://github.com/aertslab/create_cisTarget_databases; the motif collection used was created as previously described (*56*). As SCENIC is a stochastic analysis, we performed 100 runs and combined the results to identify consistent regulons. For each run, we first used the script arboreto_with_multiprocessing.py provided with pySCENIC to create adjacencies, next, pyscenic add_cor was used to compute the correlation values, followed by pyscenic ctx with the option --mode “custom_multiprocessing” to generate regulons and finally pyscenic aucell to get regulon activity scores. To limit the required computation time, we first performed 20 individual runs of SCENIC using a list containing all transcription factors present in the genome. After this, we performed a second round of 80 runs using only transcription factors that had a regulon identified in the first 20 runs. Following completion of all 100 runs, the outputs were combined into a single set of regulons using the scripts provided by vsn-pipelines [https://doi.org/10.5281/zenodo.5751297], available here: https://github.com/vib-singlecell-nf/scenic/tree/master/src/scenic/bin. In short, aggregate_multi_runs_regulons.py was run, followed by convert_multi_runs_features_to_regulons.py and save_multi_runs_to_loom.py, both with the --min-regulon-gene-occurrence 20, providing a single unified set of regulons each of which only containing target genes found in at least 20/100 runs.

### Gene set enrichment, module score and Spearman’s rank correlation analyses

Gene set enrichment analysis (GSEA) was performed in R (version 4.5.2) using the fgsea package. Pseudobulk expression profiles were generated by aggregating raw UMI counts per gene across microglia cells, grouped by condition, biological replicate, experimental batch (date), and microglial state annotation. This resulted in 2–4 pseudobulk samples per animal, with 3–4 animals per condition across three conditions. Differential expression analysis was performed using DESeq2, fitting negative binomial models. For each microglial state (Micro-A, Micro-C, Micro-T, and Micro-D), a binary comparison was performed against all other microglial states by constructing state-specific indicator variables (e.g., Micro-A vs. Micro-other). Models were fit, regressing out experimental batch effects using the design formula: ∼ date + state_indicator. Log2 fold changes were estimated using Wald tests and subsequently shrunk using the apeglm method. Genes were ranked based on the shrunken log2 fold changes for downstream enrichment analysis. Marker gene sets representing previously published microglial states were obtained from their respective studies. Mouse or human marker genes were mapped to Syrian hamster orthologs using Ensembl BioMart (accessed May 18, 2026), retaining the top-ranked ortholog per gene. GSEA was performed separately for each microglial state using the fgsea() function with 100,000 permutations (nPermSimple = 100000).

Module scores were calculated to quantify the expression of published microglial marker gene sets at the single-cell level. Marker genes were mapped to hamster orthologs as described above. Scores were computed using the AddModuleScore function implemented in the Seurat package.

To assess concordance between published marker signatures and the observed transcriptional changes, Spearman’s rank correlation analysis was performed in R (version 4.5.2). For each marker set, published log2 fold changes were matched to the corresponding hamster orthologs. These were combined with the shrunken log2 fold changes derived from pseudobulk DESeq2 analysis for each microglial state. Spearman’s rank correlation coefficients were calculated between published and observed log2 fold changes using the cor.test() function in R together with *P* values.

### Western blotting

Animals were anaesthetized with an intraperitoneal injection of pentobarbital and decapitated. Hippocampi were dissected out of the brain and homogenized in RIPA Lysis and Extraction Buffer (Thermo Fisher Scientific) with a Dounce homogenizer. Samples were lysed for 30 minutes at 4°C with rotation and lysates were centrifuged for 15 minutes at 4°C and 14,000 rpm to pellet debris. Protein concentration was measured in cleared lysates using Bradford Assay (Bio-Rad) and samples were boiled in Laemmli Sample Buffer (Bio-Rad) for 5 minutes at 95°C. Equal amounts of protein samples were loaded in NuPAGE Novex 4–12% Bis-Tris gels (Invitrogen Novex) for protein separation by electrophoresis performed in MOPS SDS Running Buffer (Invitrogen Novex). Proteins were transferred to nitrocellulose membranes (Bio-Rad) with Trans-Blot Turbo Transfer system (Bio-Rad). Membranes were washed in TBS containing 0.1% Tween 20 (TBS-T) and blocked in TBS-T with 5% milk powder (blocking solution). Membranes were subsequently incubated with primary antibodies in blocking solution overnight at 4°C, washed in TBS-T, incubated with HRP-conjugated secondary antibodies in blocking solution for one hour at room temperature and washed again in TBS-T. Membranes were incubated with Western Lightning Plus-ECL (Perkin Elmer) and protein bands were visualized by chemiluminescence and imaged in an iBright 1500 instrument (Invitrogen). Images were analyzed and processed using iBright Analysis Software (Invitrogen). The following antibodies were used: Lin54 (AB_ 3421257); Rpl24 (AB_2181728); Rpl36 (AB_2878111); Rpl36a (AB_2238666); Rps3a (AB_2253921); Rps11 (AB_2620285); Tau (AB_10013724); pTau-AT8 (AB_223647).

### Immunofluorescence and imaging

Animals were perfused with PBS and the brain was fixed by overnight incubation in 4% paraformaldehyde-containing PBS at 4°C. Coronal sections were cut with a vibratome (Leica VT1000 S) and preserved in sodium azide-containing PBS at 4°C. Sections were permeabilized in PBS containing 0.05% Triton X-100 for 10 minutes and blocked in PBS containing 0.5% Triton X-100 and 5% normal goat serum (blocking solution) for 2 hours. Subsequently, sections were incubated with primary antibodies in blocking solution overnight at 4°C, washed in PBS and incubated with secondary antibodies in blocking solution for 2 hours at room temperature. Finally, sections were washed in PBS, incubated in PBS containing 0.1 μg/mL Dapi for 5 minutes and washed again in PBS, and coverslips were mounted on top of the sections with Mowiol (Merck). Imaging was performed at the VIB-KU Leuven Center for Brain and Disease Research Light Microscopy Expertise Unit. Overview images of the hippocampus were acquired with a Nikon spinning-disk microscope with a 10X/0.5 N.Astarte lens. Images of the CA3 region were acquired with a Nikon A1R confocal microscope with a 20X/1.0 N.A. lens or a 60X/1.0 N.A. lens. Images were analyzed and processed using Fiji software (National Institutes of Health). The following antibodies were used: Gfap (AB_10013382); cFos (AB_3676416); Iba1 (AB_2924932); β-amyloid-6E10 (AB_2564652).

### RNA Scope in situ hybridization

The brains of euthermic, torpor and arousal hamsters were fixed by overnight incubation in 4% paraformaldehyde (PFA)-containing PBS at 4°C and cryoprotected by immersion in a sucrose gradient (10%, 20% and 30% sucrose-containing PBS) until they sank. Brains were frozen in Tissue-Tek OCT (Sakura Finetek), and 18 μm-thick coronal sections were cut with a NX70 Cryostat and mounted on SuperFrost Ultra Plus adhesion slides (Thermo Fisher). Sections were stained using the BaseScope Duplex Fluorescent Reagent Kit (ACD), according to the instructions provided by the manufacturer. Briefly, slides were further post-fixed for 20 minutes in 4% PFA-containing PBS following ethanol dehydration (50%, 70%, 100%). Sections were treated with hydrogen peroxide for 10 minutes and target retrieval was performed for 15 minutes at 100°C. Afterwards, sections were treated with Protease III for 30 minutes and hybridized with BaseScope probes for 2 hours at 40°C in the HybEZ II oven. Probes were fluorescently labelled using the TSA Fluorophores TSA Vivid 520 and TSA Vivid 570. Finally, RNAscope in situ hybridization was combined with immunofluorescence and imaging was performed as described in the previous section.

### Statistical analysis

Statistics used for the analysis of the single nuclei data are explained in the previous sections. For statistical analyses of the immunofluorescence or western blotting experiments, datasets were tested for normality using Shapiro-Wilk test. When datasets passed the normality test they were analyzed using one-way ANOVA with Tukey’s multiple comparisons test. In all cases, *P* values and *N* numbers are shown in the graphs and figure legends. For the specific statistical parameters of Shapiro-Wilk, ANOVA and Tukey’s test, see table S1. The *N* values correspond to the number of animals used for a specific experiment, unless otherwise specified.
