# Supplementary material for: Common transcriptional changes in the brain during hibernation and Alzheimer’s disease
Source: Sci Adv. 2026 Nov 4;12(45):eaeg3486. doi: 10.1126/sciadv.aeg3486 (PMC13733825; doi:10.1126/sciadv.aeg3486)
Supplement: Supplementary file 1 [file sciadv.aeg3486_sm.pdf]

Science Advances
AAAS

Supplementary Materials for

Common transcriptional changes in the brain during hibernation and Alzheimer's disease

Pablo Largo-Barrientos *et al.*

Corresponding author: Pablo Largo-Barrientos, pablo.largobarrientos@kuleuven.be;
Roman Praschberger, roman.praschberger@i-med.ac.at; Patrik Verstreken, patrik.verstreken@kuleuven.be

Sci. Adv. **12**, eaeg3486 (2026)
DOI: 10.1126/sciadv.aeg3486

The PDF file includes:

Figs. S1 to S12
Legends for tables S1 to S3

Other Supplementary Material for this manuscript includes the following:

Tables S1 to S3

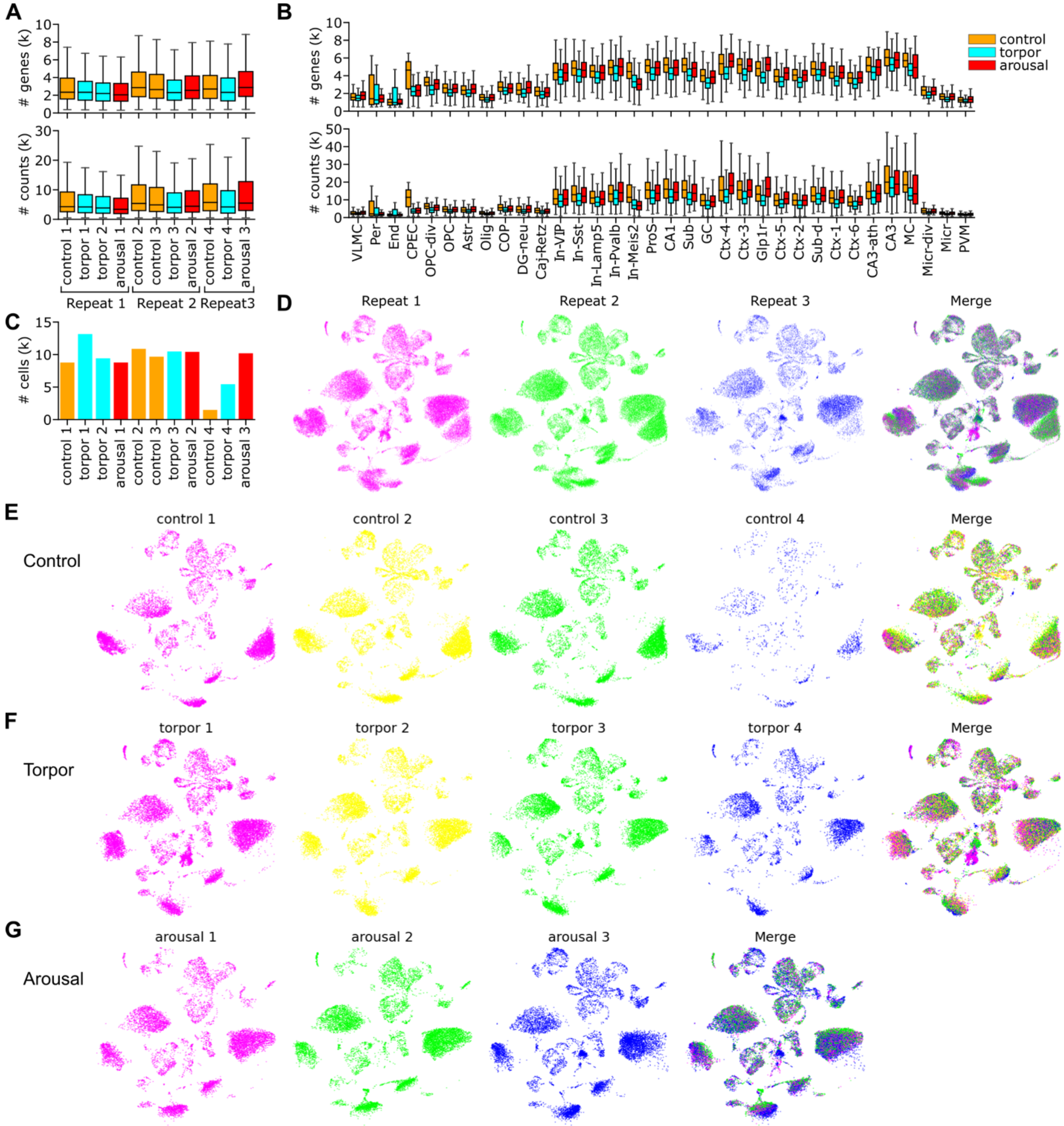

Fig. S1. Quality analysis of the single-nucleus RNA sequencing samples. (A) Number of genes and counts for each of the 11 samples that were sequenced. **(B)** Number of genes and counts for each of the 34 identified cell types in each of the three conditions (control, torpor and arousal). **(C)** Number of cells that were sequenced for each of the 11 samples. **(D)** UMAP plots depicting all the cells that were sequenced in each of the three experimental repeats. **(E-G)** UMAP plots depicting the cells that were sequenced in each individual sample for each condition.

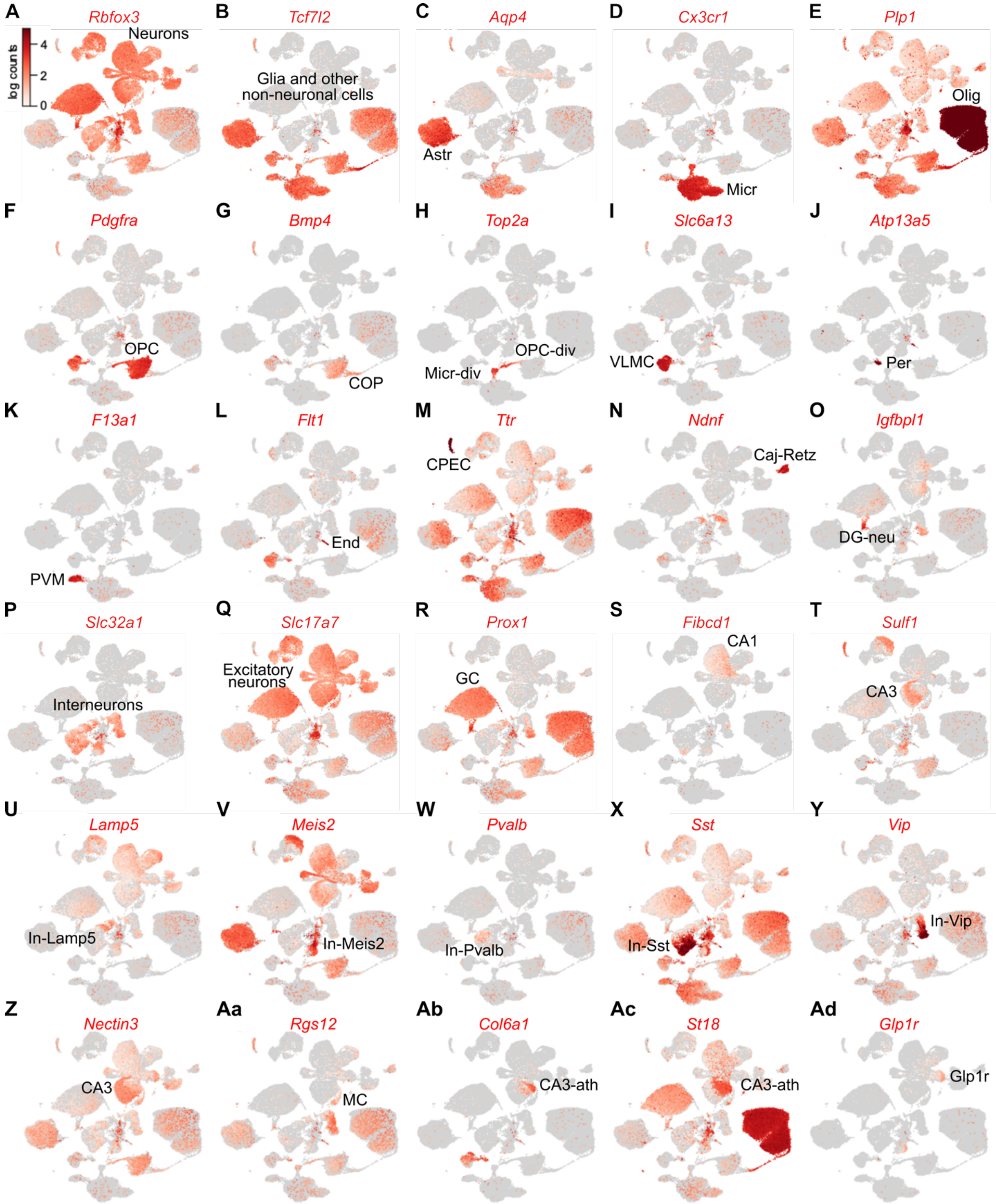

Fig. S2. Markers for the cell clusters. (A-Ad) UMAP plot of the expression of markers for the different cell types identified in the hippocampus of the golden hamster (Astr, astrocytes; Micr, microglia; Olig, oligodendrocytes; OPC, oligodendrocyte precursor cells; COP, committed oligodendrocyte precursor cells; OPC-div, dividing oligodendrocyte precursor cells; Micr-div, dividing microglia; VLMC, vascular and lepotomeningeal cells; Per, pericytes; PVM, perivascular macrophages; End, endothelial cells; CPEC, choroid plexus epithelial cells; Caj-Retz, Cajal-Retzius cells; DG-neu, neuronal progenitor cells or immature granule cells from the subgranular zone of the dentate gyrus; GC, granule cells; CA1, CA1 pyramidal neurons; CA3, CA3 pyramidal neurons;

In-Lamp5, Lamp5 interneurons; In-Meis2, Meis2 interneurons; In-Pvalb, Parvalbumin interneurons; In-Sst, Somatostatin interneurons; In-VIP, VIP interneurons; MC, mossy cells; CA3-ath, CA3 athorny pyramidal neurons; Glp1r, Glp1r-expressing neurons).

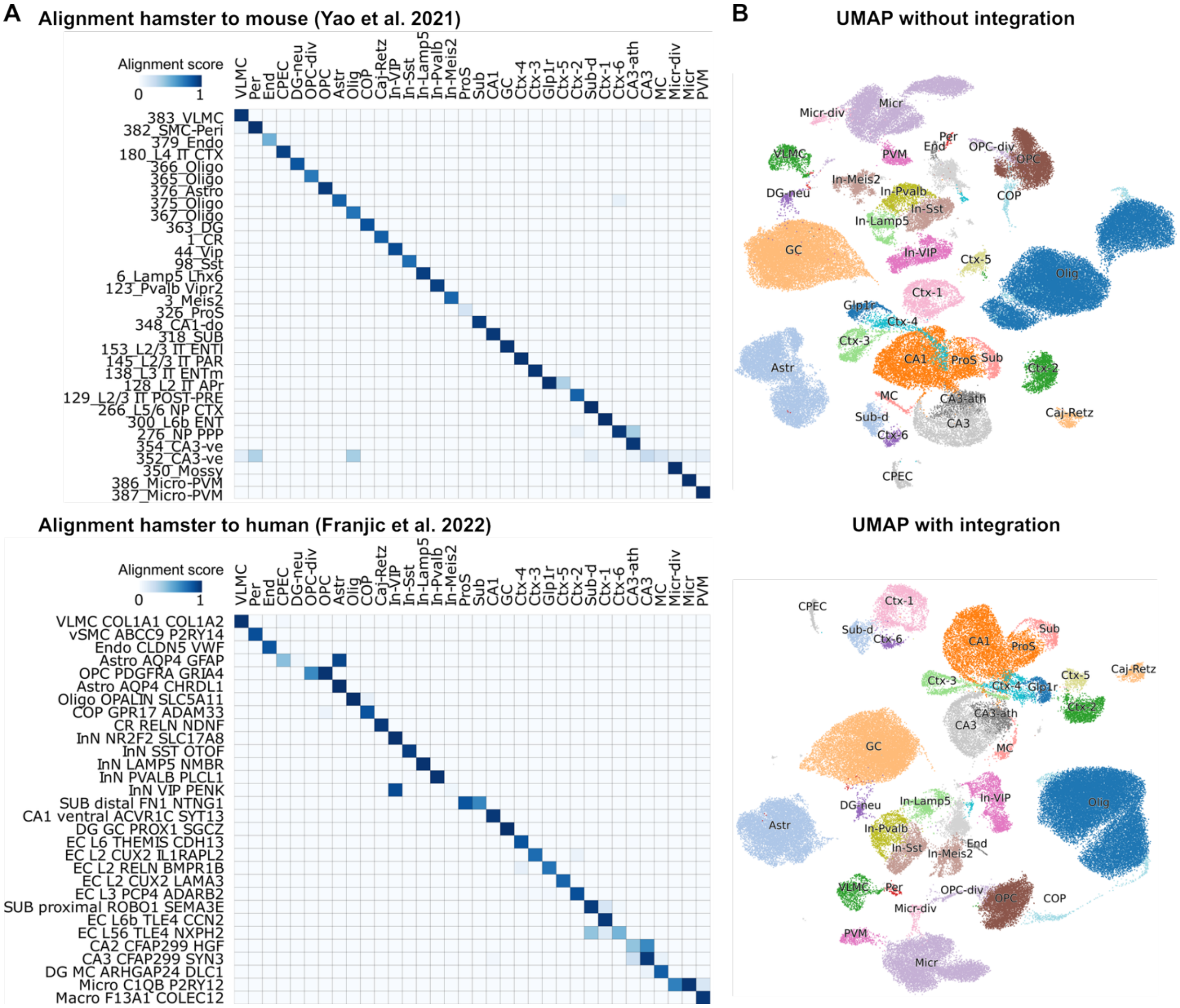

Fig. S3. Validation of the cell clustering. (A) Graphs depicting the SAMap alignment score for each of the 34 identified hamster cell clusters with the cell types identified in previous single-cell atlases from mouse and human brain. The top aligning cell types from these datasets are shown. **(B)** UMAP plots depicting hamster hippocampal cell types described in Fig. 1, with and without using Harmony for integration.

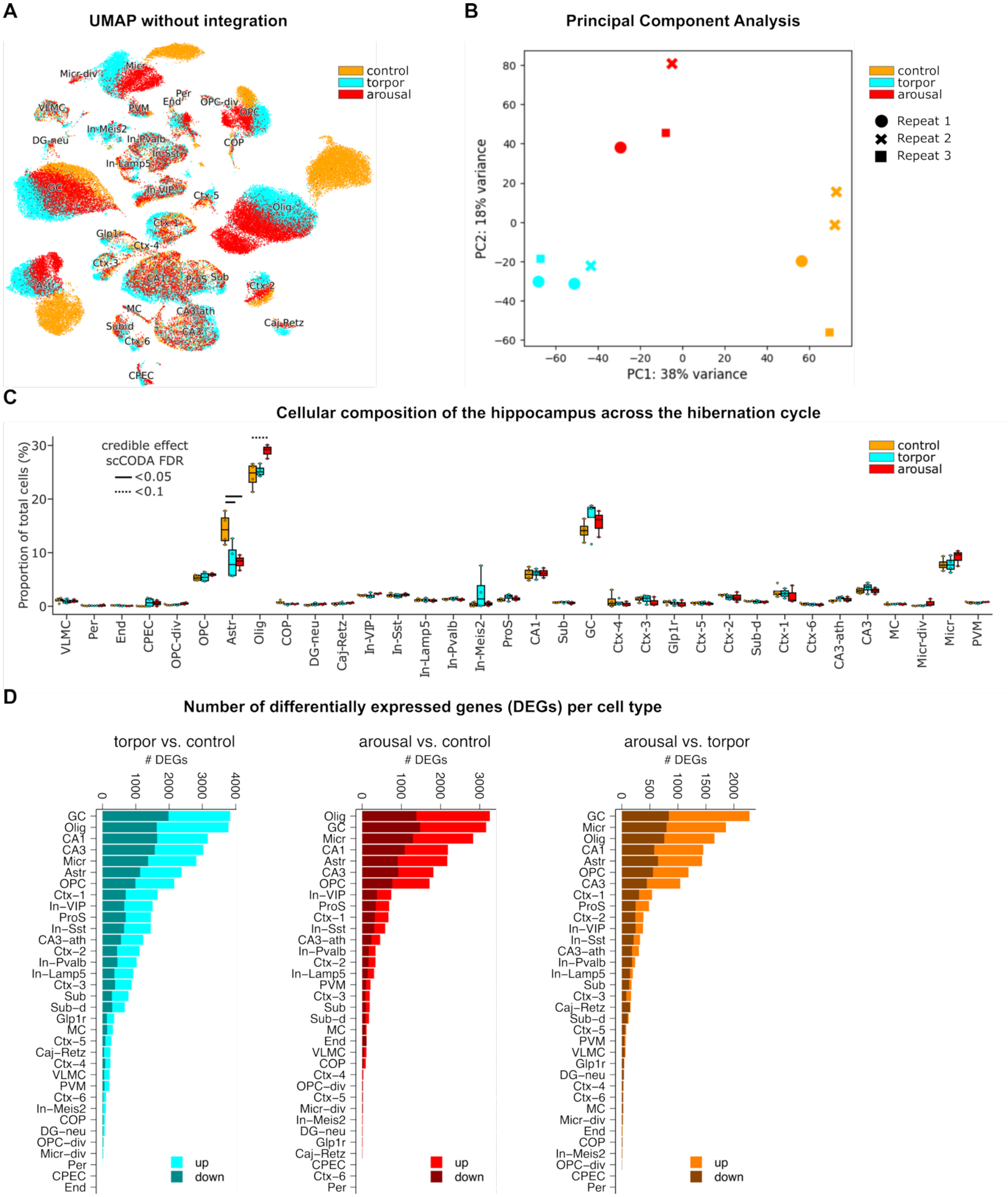

Fig. S4. Hibernation-induced changes in the hippocampus. (A) Principal component analysis plot depicting the 11 samples that were sequenced, labelled according to their group (control, torpor and arousal) and experimental repeat (date). **(B)** UMAP plot depicting hippocampal cells from hamsters within different hibernation states, without using Harmony for integration. **(C)** Cellular composition of the hippocampus of hamsters in control, torpor and arousal. Graph depicts the percentage of total cells represented by each cell type in each repeat. Testing for credible effects was conducted with scCODA. **(D)** Number of DEGs in torpor (compared to control) and arousal (compared to control and torpor) in each of the different cell types at FDR<0.05 and an absolute log-fold change higher than 0.5.

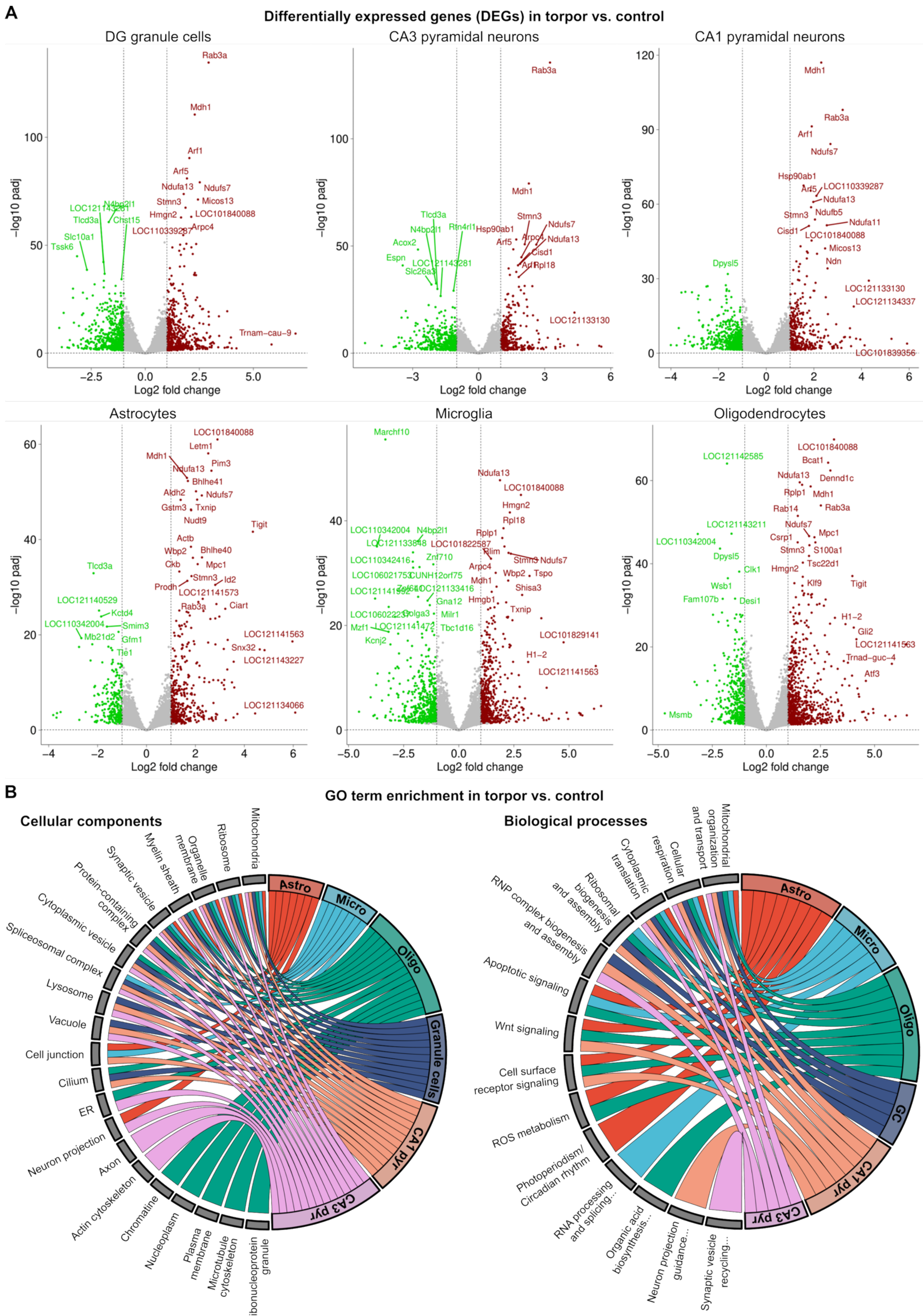
A
Differentially expressed genes (DEGs) in torpor vs. control
DG granule cells
CA3 pyramidal neurons
CA1 pyramidal neurons
Astrocytes
Microglia
Oligodendrocytes
−log10 padj
Log2 fold change
Rab3a
Mdh1
Arf1
Arf5
Ndufa13
Ndufs7
Stmn3
Micos13
Hmgn2
LOC101840088
Arpc4
LOC110339287
Trnam−cau−9
Tlcd3a
N4bp2l1
Chst15
Slc10a1
Tssk6
Hsp90ab1
Rtn4rl1
Acox2
Espn
Slc26a3
LOC121143281
Cisd1
Rpl18
LOC121133130
Ndufb5
Ndufa11
Ndn
Dpysl5
LOC121134337
LOC101839356
Letm1
Pim3
Bhlhe41
Aldh2
Gstm3
Txnip
Nudt9
Tigit
Actb
Wbp2
Bhlhe40
Ckb
Mpc1
Prodh
Id2
LOC121141573
Ciart
LOC121140529
Kctd4
Smim3
LOC110342004
Mb21d2
Gfm1
Tle1
LOC121141563
Snx32
LOC121143227
LOC121134066
Marchf10
Hmgn2
Rplp1
LOC101822587
Rlim
Tspo
Shisa3
Hmgb1
Gna12
Milr1
Tbc1d16
Mzf1
Kcnj2
LOC101829141
H1−2
LOC121142585
Bcat1
Dennd1c
Rab14
Csrp1
S100a1
Tsc22d1
Klf9
Clk1
Wsb1
Fam107b
Desi1
Gli2
Trnad−guc−4
Atf3
Msmb
B
GO term enrichment in torpor vs. control
Cellular components
Mitochondria
Ribosome
Organelle membrane
Myelin sheath
Synaptic vesicle
Protein-containing complex
Cytoplasmic vesicle
Spliceosomal complex
Lysosome
Vacuole
Cell junction
Cilium
ER
Neuron projection
Axon
Actin cytoskeleton
Chromatine
Nucleoplasm
Plasma membrane
Microtubule cytoskeleton
Ribonucleoprotein granule
Astro
Micro
Oligo
Granule cells
CA1 pyr
CA3 pyr
Biological processes
Mitochondrial organization and transport
Cellular respiration
Cytoplasmic translation
Ribosomal biogenesis and assembly
RNP complex biogenesis and assembly
Apoptotic signaling
Wnt signaling
Cell surface receptor signaling
ROS metabolism
Photoperiodism/ Circadian rhythm
RNA processing and splicing...
Organic acid biosynthesis...
Neuron projection guidance...
Synaptic vesicle recycling...
GC

Fig. S5. DEGs in torpor. (A) Volcano plots depicting the DEGs in torpor (compared to control) in the six main neuronal and glial cell types. Only genes with an adjusted P value lower than 0.05 and a log-fold change higher than 0.5 (or lower than -0.5) are coloured in the graphs (red, upregulated genes; green, downregulated genes). **(B)** Chord diagram depicting gene ontology (GO) terms enriched in torpor (compared to control) in the six major neuronal and glial cell types. Highly similar terms were grouped for clarity. For cellular components: mitochondria (GO:0005743; 0098798; 0098800; 0098803; 1990204; 0070469; 0005746; 0005747; 0030964; 0045271; 0032592; 0098573; 0031966; 0031305; 0031304; 0045255; 0005753; 0005741; 0019867; 0031968; 0000276); ribosome (GO:0005840; 0022626; 0044391; 0022625; 0022627; 0015934; 0015935; 0005844; 0042788; 0000313; 0005761; 0000315; 0005762); organelle membrane (GO:0019866; 0031300; GO0031301; 0019898; 0031225); synaptic vesicle (GO:0008021; 0098993; 0098563; 0030672); cytoplasmic vesicle (GO:0099501; 0030658; 0012506; 0030659; 0070382; 0030137; 0030135; 0030133; 0005798; 0030141; 0099503); protein-containing complex (GO:0016469; 0033177; 0033176; 0045263; GO:0000502; 0022624; 0005838; GO:0031248; 1902493; GO:1905368; 1905369); spliceosome complex (GO:0005681; 0071013; 0005684; 0005686); vacuole (GO:0005773; 0005774; 0000323; 0016471); cell junction (GO:0005925; 0070161; 0030055; 0005911; 0005923); cilium (GO:0097225; 0036064); ER (GO:0005789; 0005791); neuron projection (GO:0044306; 0032420); axon (GO:0150034; 0043679); chromatin (GO:0070603; 0000792; 0016581; 0090545; 0000123); nucleoplasm (GO:0035097; 0000118); plasma membrane (GO:0016342; 0019897); microtubule cytoskeleton (GO:0000930; 0005814). For biological processes: mitochondrial organization and transport (GO:0007005; 0006839; 0010257; 0032981; 1990542; 0007006; 0007007; 0070585); cellular respiration (GO:0045333; 0015980; 0006091; 1902600; 0042773; 0006119; 0046034; 0022900; 0042775; 0022904; 0009060; 0006099); ribosome biogenesis and assembly (GO:0042254; 0042255; 0042273; 0042274; 0000027; 0000028); ribonucleoprotein (RNP) complex biogenesis and assembly (GO:0022613; 0022618; 0071826); apoptotic signalling pathway (GO:0097193; 1902229; 2001242; 2001244); Wnt signaling (GO:0198738; 0016055; 0030111; 0060070; 0030177; 0030178); circadian rhythm (GO:0009649; 0007623; 0043153); RNA processing and splicing (GO:0008380; 0006364; 0016072; 0000375; 0000377; 0000398; 0006397; 0034470; 1903312); organic acid biosynthesis (GO:0016053; 0046394). Not included in the diagram: membrane depolarization (GO:0051899; 0003254; 1904181); mitochondrial depolarization (GO:0051881; 0051882; 0051900), small GTPase mediated signal transduction (GO:0007264; 0007265; 0007266); translational elongation, only in microglia. Antimicrobial humoral response (GO:0019730; 0061844); peptydil-lysine modification, chromatin modification, histone modification, organelle assembly and cilium assembly, only in oligodendrocytes. Regulation of neurotransmitter receptor activity, neuron projection guidance, regulation of cell morphogenesis, cell-substrate adhesion, taxis/chemotaxis, protein autophosphorylation and Golgi vesicle transport, only in CA1 pyramidal neurons. Vesicle localization (GO:0051648; 0051650); catecholamine secretion (GO:0051937; 0050433); regulation of amine transport, regulation of protein polymerization, regulation of actin polymerization and regulation of protein-containing complex assembly, only in CA3 pyramidal neurons.

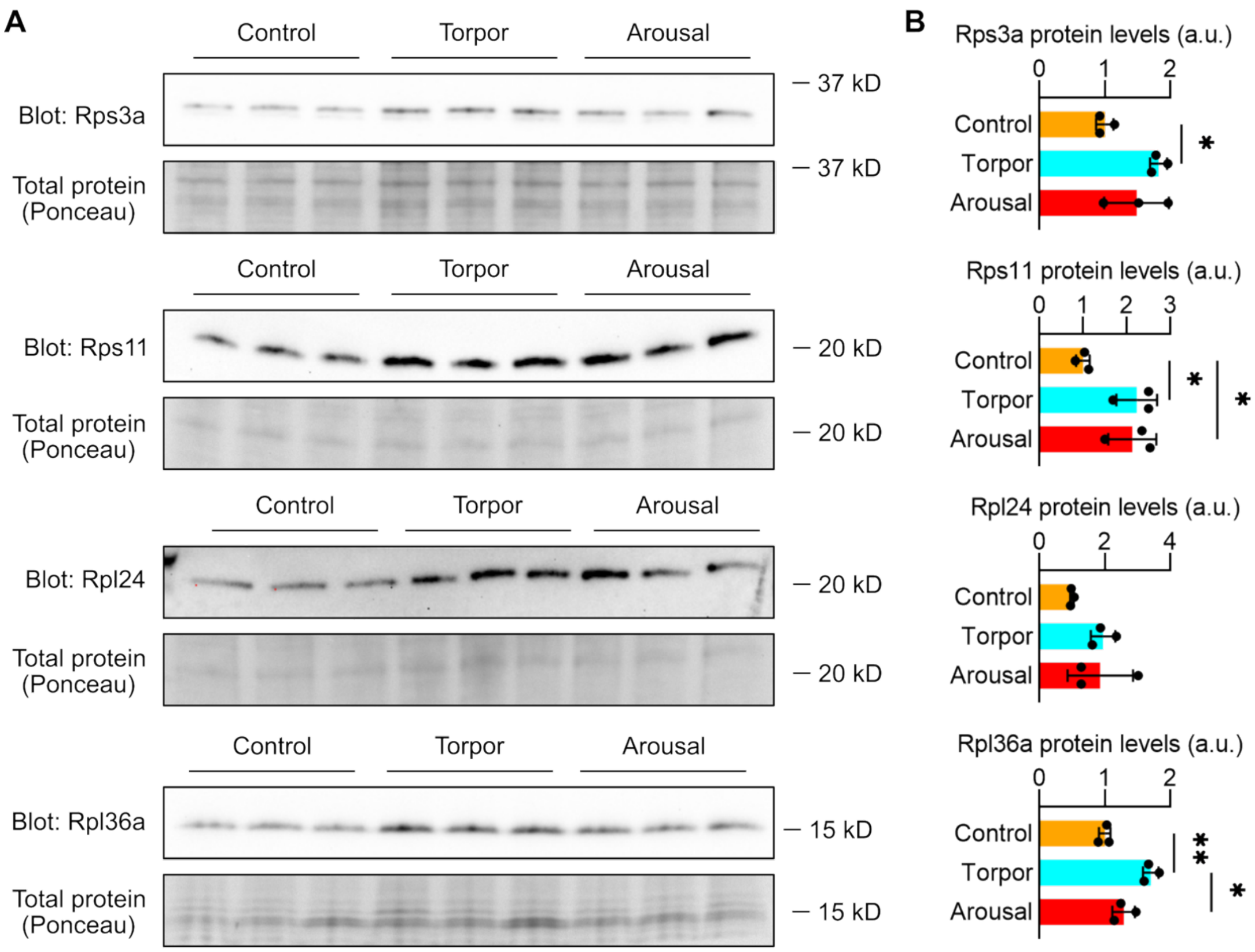

Fig. S6. Upregulation of ribosomal proteins in torpor. (A) Western blots showing Rps3a, Rps11, Rpl24 and Rpl36a protein levels in hippocampal homogenates from control, torpor and arousal hamsters. **(B)** Quantification of A. Graphs depict mean ± SD (*P < 0.05). Detailed statistical results are provided in Table S1.

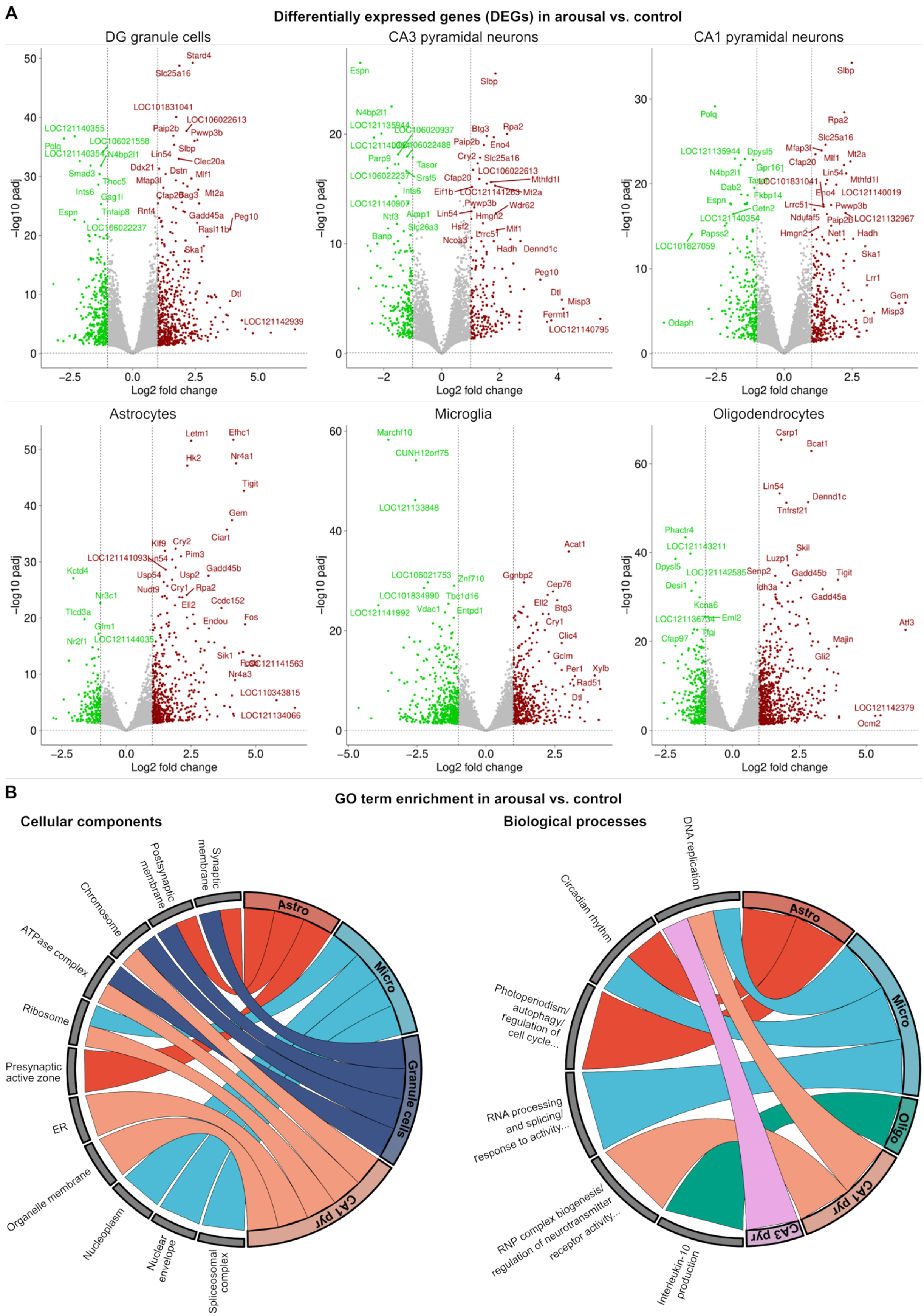

Fig. S7. DEGs in arousal. (A) Volcano plots depicting the DEGs in arousal (compared to control) in the six main neuronal and glial cell types. Only genes with an adjusted P value lower than 0.05 and a log-fold change higher than 0.5 (or lower than -0.5) are coloured in the graphs (red,

upregulated genes; green, downregulated genes). **(B)** Chord diagram depicting gene ontology (GO) terms enriched in arousal (compared to control) in the six major neuronal and glial cell types. Highly similar terms were grouped for clarity. For cellular components: synaptic membrane (GO:0099240; 0099699); postsynaptic membrane (GO:0045211; 0098839; 0098936; 0099055; 0099060; 0099061; 0099146; 0099634); chromosome (GO:0000775; 0000776; 0098687); ribosome (GO:0005840; GO:0022626; GO:0044391); ER (GO:0030176; 0031227); organelle membrane (GO:0031300; 0031301); nuclear envelope (GO:0005635; 0031965). For biological processes: DNA replication (GO:0006260; 0006275); circadian rhythm (GO:0007622; 0007623; 0032922; 0042752; 0043153; 0048511; 0048512; 0009649); autophagy (GO:0006914; 0061919); RNA processing and splicing (GO:0000375; 0000377; 0000398; 0006397; 0008380); Interleukin-10 production (GO:0032613; 0032653). Not included in the diagram: mitochondrial organization and transport (GO:0006839; GO:0007006); regulation of catabolic process (GO:0009896; 0031331); response to peptide (GO:1901652; 1901653); response to hormone (GO:0009725; 0032870; 0043434; 0071375), response to peptide, response to radiation, regulation of catabolic process, monocarboxylic acid metabolism, cilium assembly and glutamate receptor signaling pathway, only in astrocytes. Nucleic acid transport (GO:0006405; 0015931; 0050657; 0050658; 0051028; 0051236); RNA processing and splicing, RNA metabolism, lactate metabolism, response to activity, regulation of viral process, protein ubiquitination, hemostasis and small GTPase mediated signal transduction, only in microglia. Protein localization to cilium (GO:0061512; 0097499), only in CA1 pyramidal neurons.

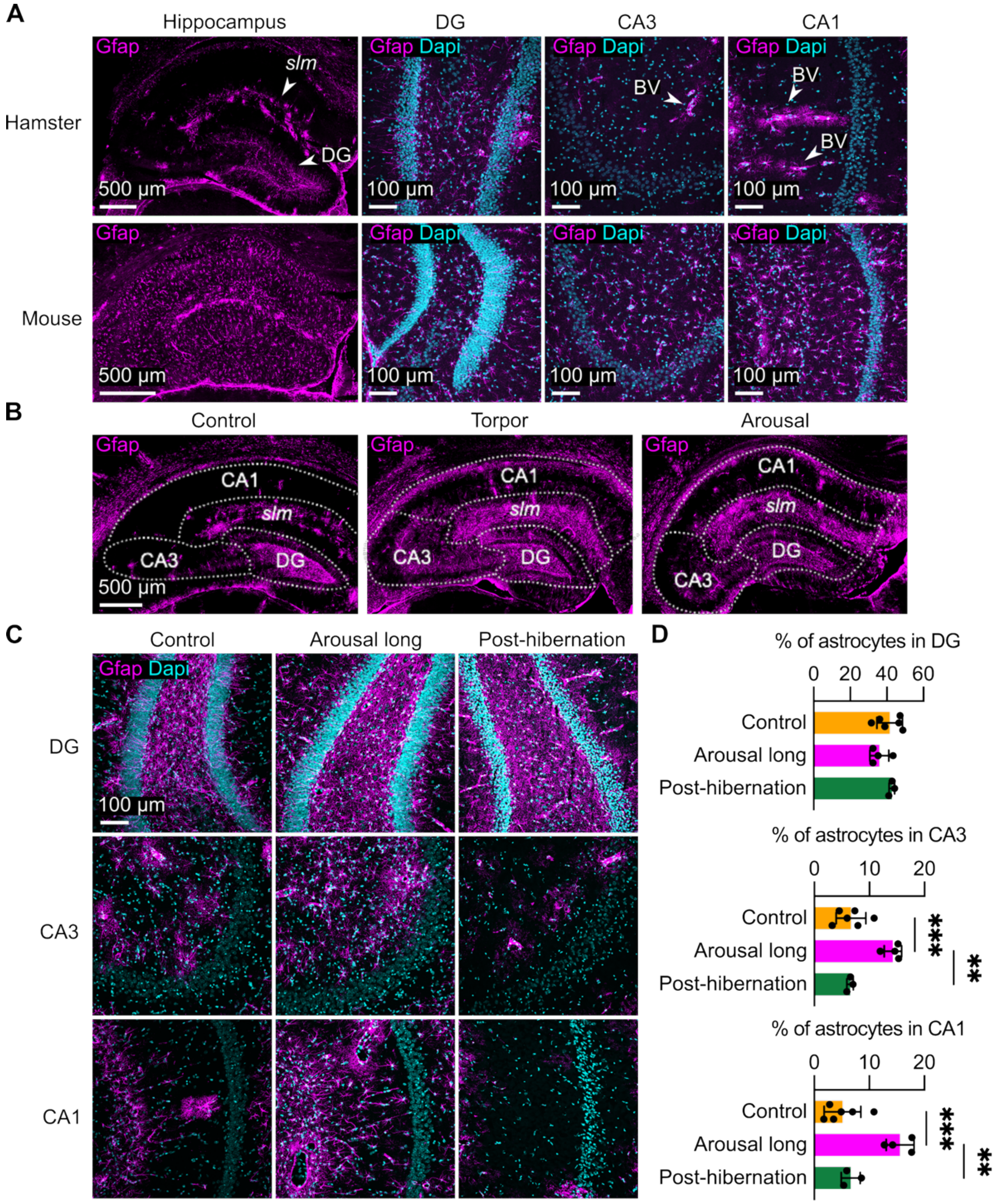

Fig. S8. Hamster astrocytes across the hibernation cycle. (A) Representative confocal images of astrocytes from the hippocampus of a golden hamster and a mouse labelled with astrocytic marker Gfap and nuclear marker Dapi. DG, dentate gyrus; slm, *stratum lacunosum moleculare*; BV, blood vessels. **(B)** Representative confocal images of astrocytes from control, torpor and arousal hamsters with the delineation of the four regions used for quantification in Fig. 4G. **(C)** Representative confocal images of astrocytes from control, arousal long (24 hours) and post-hibernation hamsters. **(D)** Quantification of C. Graph depicts mean ± SD (**P < 0.01; ***P < 0.001). Detailed statistical results are provided in Table S1.

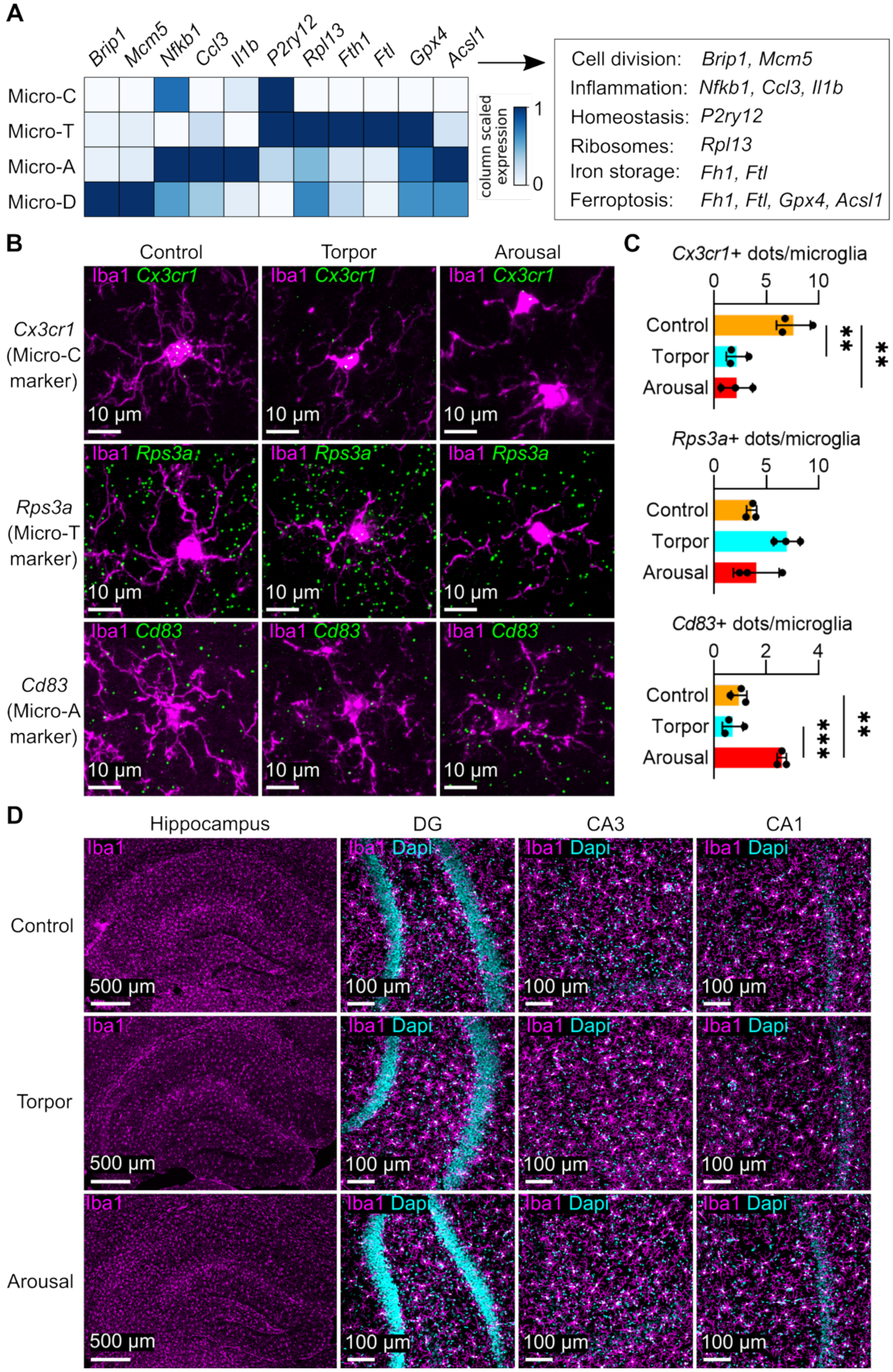

Fig. S9. Hamster microglia across the hibernation cycle. (A) Column scaled expression for selected genes in the four different subtypes of hamster microglia (Micro-C: control; Micro-T: torpor; Micro-A: arousal; Micro-D: dividing). **(B)** Representative confocal images of microglia from control, torpor and arousal hamsters labelled with microglial marker Iba1 and RNA probes (using RNAScope) for *Cx3cr1* (marker for "Micro-C"), *Rps3a* (marker for "Micro-T") and *Cd83* (marker for "Micro-A"). **(C)** Quantification of B. Graph depicts mean ± SD (**P < 0.01; ***P < 0.001). Detailed statistical results are provided in Table S1. **(D)** Representative confocal images of microglia from control, torpor and arousal hamsters labelled with microglial marker Iba1 and nuclear marker Dapi.

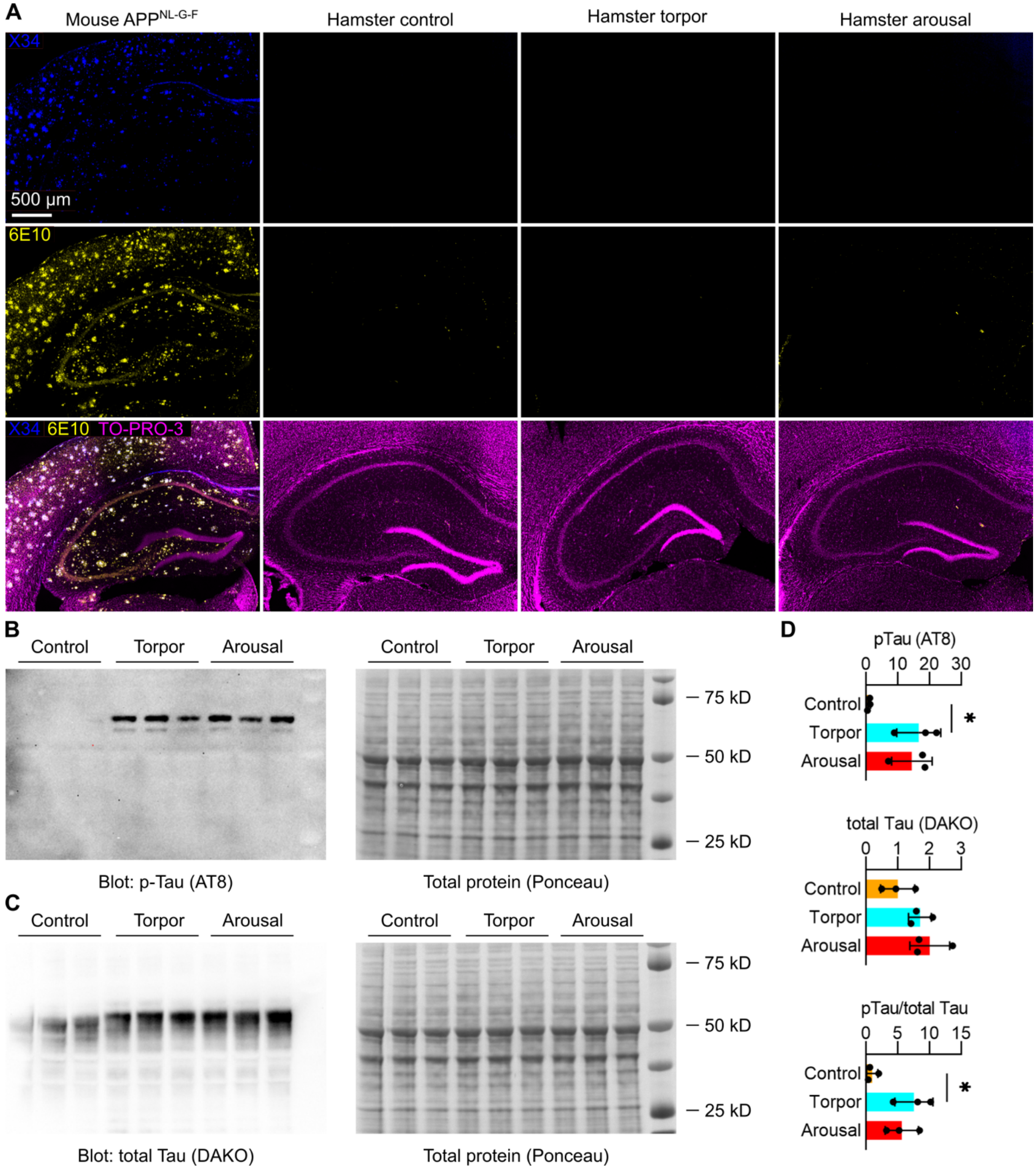

Fig. S10. Aβ and Tau in hibernation. (A) Representative confocal images of the hippocampus from an APPNL-G-F mouse and control, torpor and arousal hamsters labelled with Aβ markers X34 and 6E10 and nuclear marker TO-PRO-3. **(B,C)** Western blots showing phosphorylated Tau (AT8 antibody) and total Tau (DAKO antibody) in hippocampal homogenates from control, torpor and arousal hamsters. **(D)** Quantification of B and C. Graph depicts mean ± SD (*P < 0.05). Detailed statistical results are provided in Table S1.

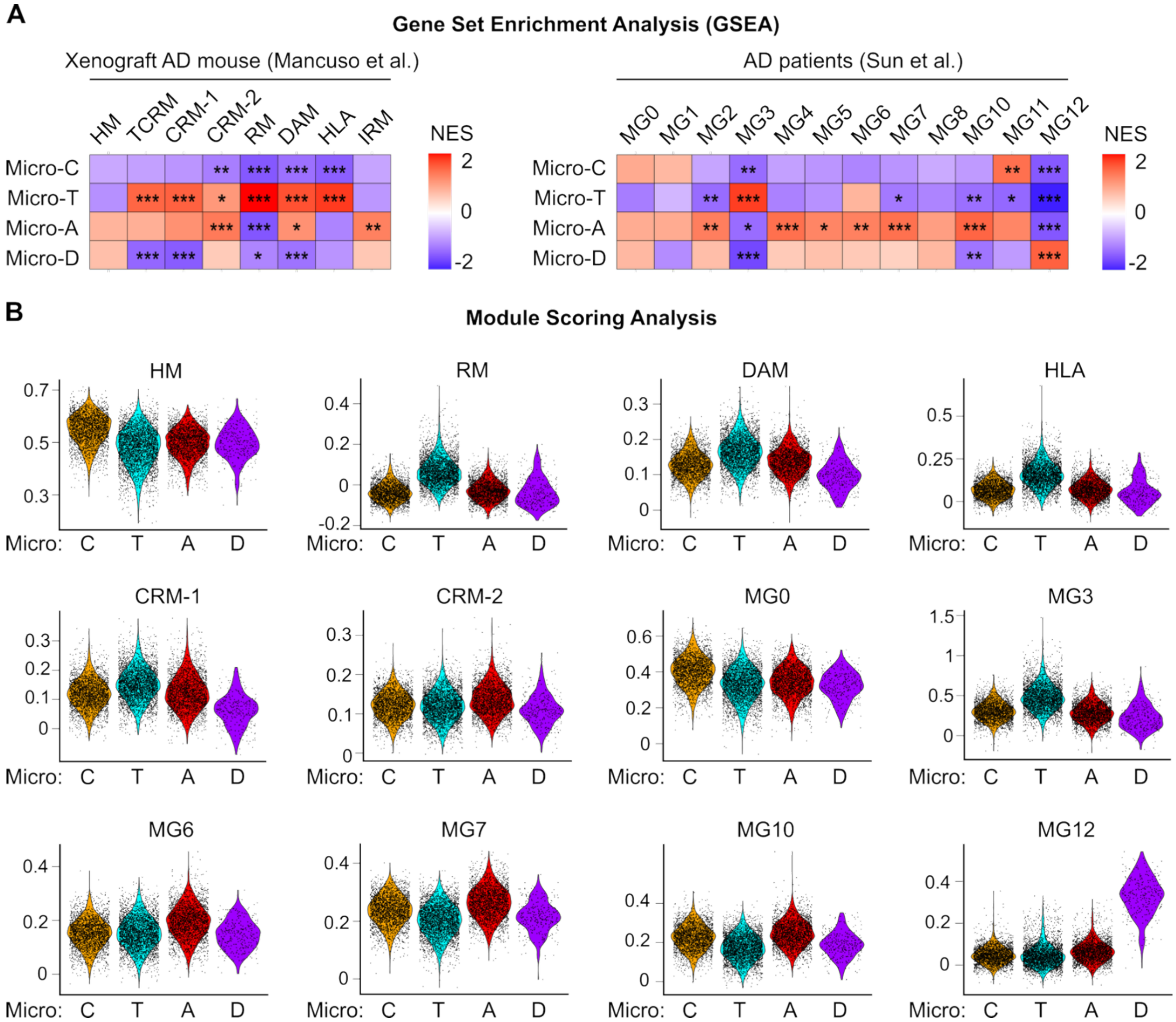

Fig. S11. Comparisons between microglial states in hibernation and Alzheimer's disease. (A) Gene set enrichment analysis (GSEA) comparing transcriptional signatures of hamster hibernation-associated microglial states (Micro-C: control; Micro-T: torpor; Micro-A: arousal; Micro-D: dividing) with published microglial states identified in a xenograft mouse AD model and in AD patients. **(B)** Module scoring calculated using published marker gene signatures from human microglial states showing the strongest association with hamster microglial states in A.

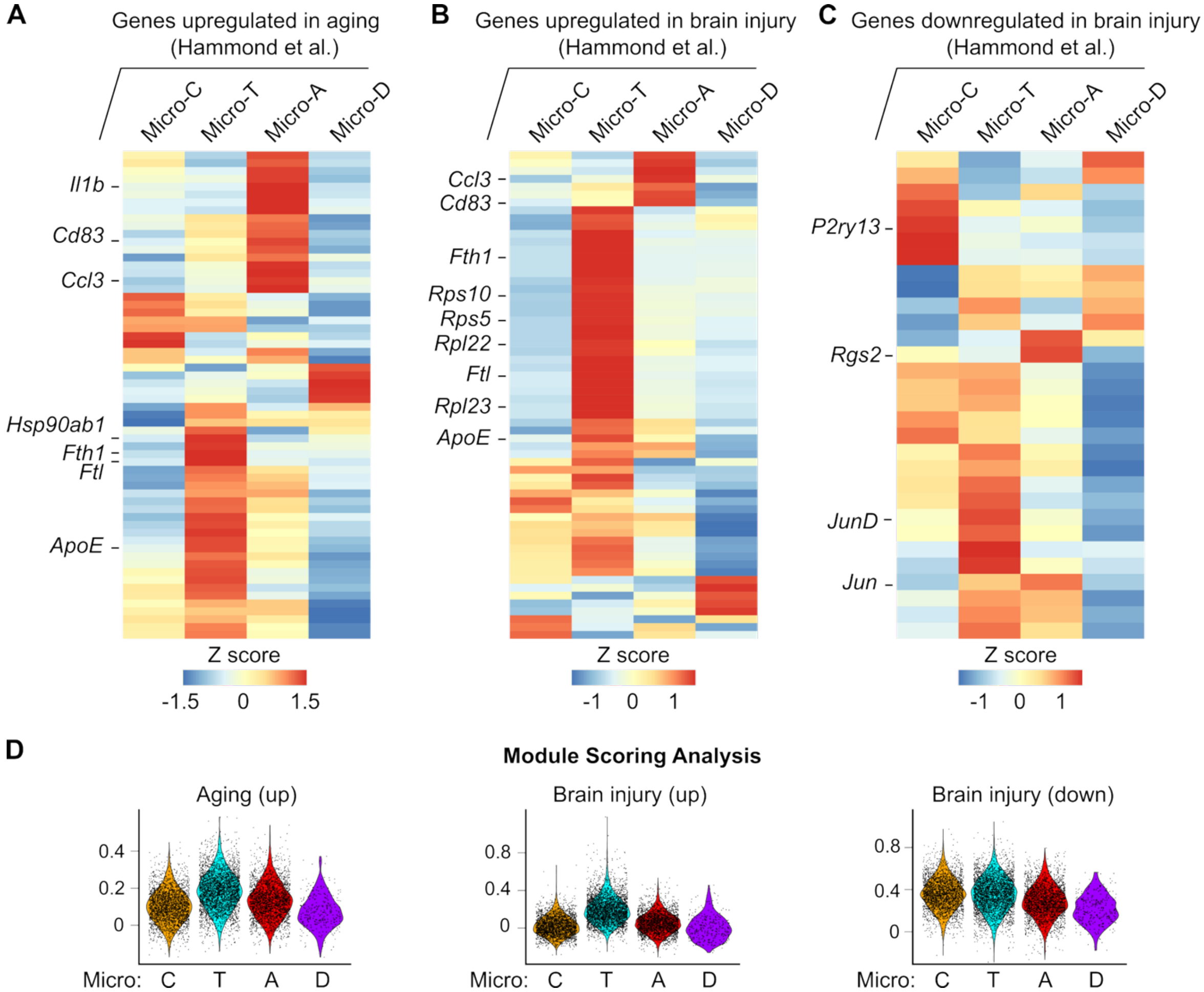

Fig. S12. Comparisons between microglial states in hibernation, aging and brain injury. (A-C) Table scaled expression for genes up- or downregulated in microglia from aging and brain injury mouse models in the four different subtypes of hamster microglia (Micro-C: control; Micro-T: torpor; Micro-A: arousal; Micro-D: dividing). **(D)** Module scoring calculated using published marker gene signatures from mouse microglial states in aging and brain injury.

Supplementary Tables (provided as Excel files)

Table S1. Statistical parameters. Summary of the parameters from all the statistical tests used in the study.

Table S2. GO term analysis in torpor. Summary of GO terms enriched in torpor (compared to control) for all 34 identified cell types.

Table S3. GO term analysis in arousal. Summary of GO terms enriched in arousal (compared to control) for all 34 identified cell types.
